# Label-free whole-colony imaging and metabolic analysis of metastatic pancreatic cancer by an autoregulating flexible optical system

**DOI:** 10.7150/thno.40869

**Published:** 2020-01-01

**Authors:** Binglin Shen, Junshuai Yan, Shiqi Wang, Feifan Zhou, Yihua Zhao, Rui Hu, Junle Qu, Liwei Liu

**Affiliations:** Key Laboratory of Optoelectronic Devices and Systems of Guangdong Province & Ministry of Education, College of Physics and Optoelectronic Engineering, Shenzhen University, Shenzhen, China.

**Keywords:** metastatic cancer, label-free imaging, nonlinear optical imaging, autofluorescence lifetime, phasor approach

## Abstract

Cancer metastasis is a Gordian knot for tumor diagnosis and therapy. Many studies have demonstrated that metastatic processes are inevitably affected by the tumor microenvironment. Histopathology is used universally as the gold standard for cancer diagnosis despite the lengthy preparation process and invasiveness.

**Methods**: Here, we introduced a supercontinuum and super-wide-tuning integrated multimodal platform, which combines the confocal, nonlinear and fluorescence lifetime microscopy with autoregulations, for label-free evaluation of fresh tissue and pathological sections. Based on various automated tunable lasers, synchronized and self-adjusting components and eight fast switching detection channels, the system features fast, large-field and subcellular-scale imaging of exogenous and endogenous fluorophores, nonlinear coherent scattering and lifetime contrast.

**Results**: With such an integrated multi-dimensional system, we searched the metastatic region by two-photon and three-photon excited autofluorescence, analyzed the cancer invasion by second harmonic generation and revealed the affected cellular metabolism by phasor-lifetime. We demonstrated the flexible measurement of multiple nonlinear modalities at NIR I and II excitation with a pre-compensation for group delay dispersion of ~7,000 fs^2^ and low power of <40 mW, and of dual autofluorescence lifetime decays for phasor approach to decompose cancer-associated and disassociated components. This significantly revealed the metastatic and metabolic optical signatures of the whole colony of pancreatic cancers.

**Conclusion**: The synergistic effect of the system demonstrates the great potential to translate this technique into routine clinical applications, particularly for large-scale and quantitative studies of metastatic colonization.

## Introduction

Cancer is a major cause of mortality for modern people. Approximately 90% of cancer-associated deaths are due to the metastasis of disseminated tumor cells to distant parts of the body where they survive to reinitiate their proliferative programs and start uncontrolled neoplastic growths 1-3. Pancreatic adenocarcinomas are among the most fatal cancers due to the extreme difficulty of early detection and their strong ability to perform local invasion into surrounding tissues and distant metastasis to other organs at an early stage, and hence have attracted a vast amount of attentions 4-7. In spite of innovative treatment strategies such as the combination of ERK (extracellular regulated protein kinase) 6 and autophagy inhibition 7, surgery (with a few approved drugs) is the only potentially curative option for these pharmacologically intractable cancers 8. Aiming to remove tumors, especially metastatic tumors, completely while minimizing morbidity and avoiding relapse, a successful surgical resection requires the exact identification of the surgical margins, which can be defined by the extent of tumor infiltration or the boundary between the normal and metastatic tissue 9, 10. The imaging techniques that are widely used in clinics, such as magnetic resonance imaging (MRI), computed tomography (CT), positron emission tomography (PET), and ultrasound (US), rely primarily on morphological criteria to discern cancer metastases 11 and have shown great accomplishments in tumor detection 10-12. However, accurate discrimination of the metastatic margins at the cellular scale remains a significant challenge, and there are a lot of mesoscopic biochemical processes, such as cellular metabolism involving endogenous NADH (nicotinamide adenine dinucleotide) and FAD (flavin adenine dinucleotide) 13, 14, which accompany the most vigorous period of metastatic tumor growth and could be used to elevate recognition accuracy of the margins, remaining insufficient in research. Although histology is well accepted as the gold standard process for cancer diagnosis, it is time-consuming, labor-intensive and inherently destructive because of staining 15. Thus, in order to acquire multidimensional information while improving the spatial and temporal resolution, as well as the specificity for clinical diagnosis and medical research, there are increasing demands for the development of novel imaging modalities that can perform noninvasive and rapid diagnoses without standard histology procedures and other fluorescent probe methods. As microscopic technology advances, we hope to change the way we detect, visualize, and monitor disease so that it can be better used for diagnosis and treatment.

**Figure 1:**
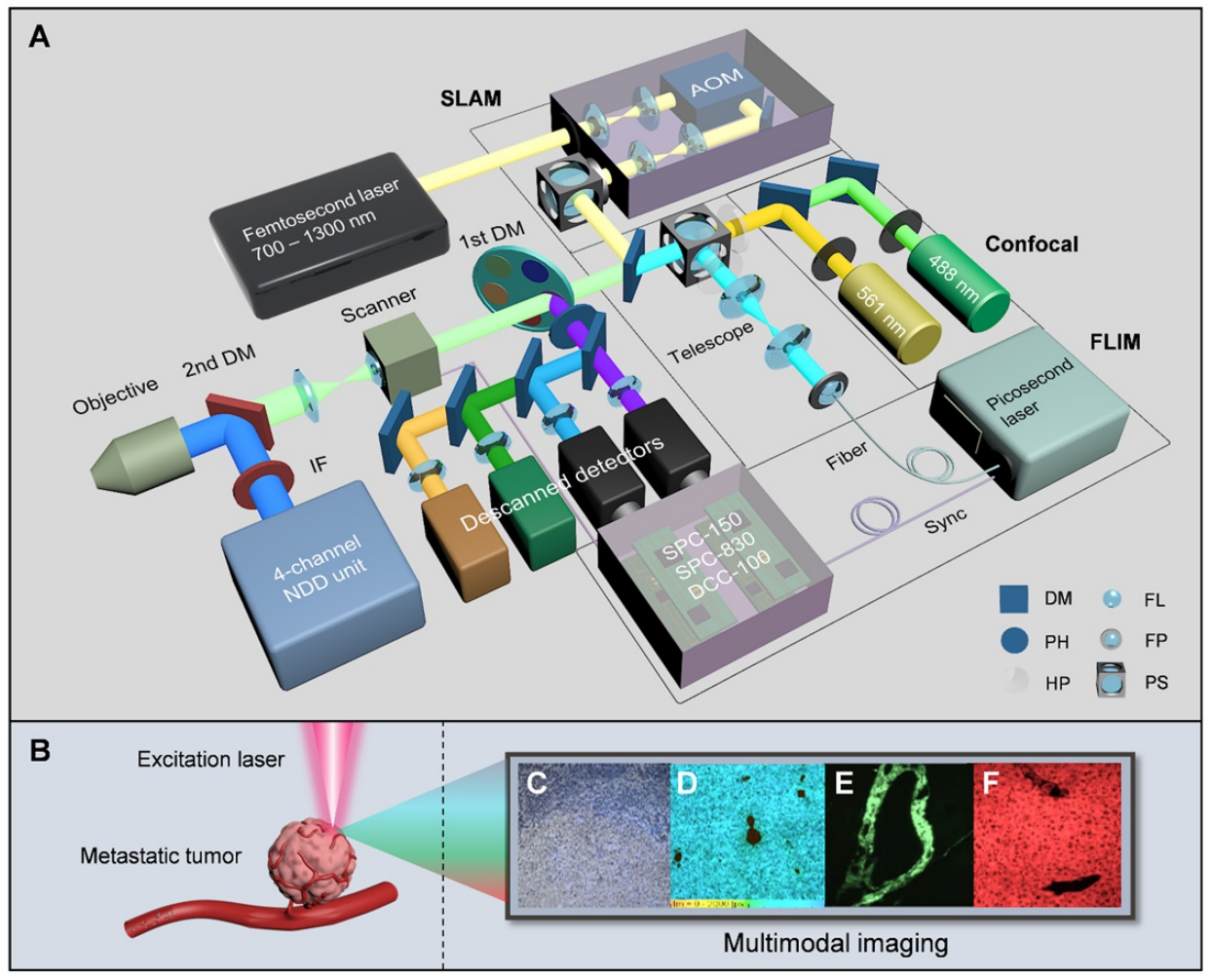
An autoregulating flexible optical system to analyze tumor microenvironment of primary and metastatic cancers. **A,** Schematic of the SIMPLE system. The double-output CW laser (488 nm and 561 nm) is coupled parasynchronously with two descanned detectors. The SLAM combines the broadly-tunable femtosecond laser (700 nm-1300 nm), the AOM-based incident optical unit and the NDD unit. The FLIM is implemented by creating a chain of synchronously interconnected components, precisely connecting the supercontinuum picosecond laser, the synchronized scanner and the time-resolved detectors to the TCSPC modules. **B,** Label-free excitation on metastatic tumor, **C**-**F,** Images from CFM (C), FLIM (D), and SLAM (E and F). AOM acousto-optic modulator, IF IR-cut filter, DM dichroic mirror, PH pin hole, HP half-wave plate, FL focal lens, FP fiber port, PS polarization splitter.

Over the past decade, nonlinear optical imaging techniques, mainly including two-photon excited fluorescence (2PEF), three-photon excited fluorescence (3PEF), second harmonic generation (SHG), third harmonic generation (THG) and coherent anti-Stokes Raman scattering (CARS), have shown great capabilities to discriminate individual specialized cell types within tumors, and could even be substituted for surgical frozen-section analysis with fast turnaround times and direct fresh tissue examination 15-18. However, the integration of various nonlinear processes typically requires different excitation bands and sequential image collection 17, and the reprogramming of energy metabolism, an important hallmark of cancer 19, cannot be completely revealed by nonlinear imaging especially before complete metastatic colonization. Fluorescence lifetime imaging microscopy (FLIM), which is the combination of fluorescence lifetime measurement and fluorescence microscopy, has emerged as a promising imaging modality for tumor environment analysis. Unlike most nonlinear imaging techniques based on qualitative analysis, FLIM can achieve quantitative analysis of biological processes in tumor microenvironment 20-22. Tremendous progress has been made toward understanding cellular energy metabolism by using FLIM 23-25. However, simultaneous implementation of more than 3 nonlinear imaging modalities by long-wavelength excitation and lifetime and component-ratio analysis by fit-free phasor approach has been lacking for a long time. To study the tumor microenvironment formed during the course of metastatic tumorigenesis 19, complementary contrast mechanisms should be combined, including the fluorescence intensities and lifetimes of NADH and FAD, which are natural biomarkers of cellular metabolism 26-29, and the scattering signals of collagen fibers and optical heterogeneity, which are large products of matrix reorganization in cancerization 30-32 and indicators of a nonhomogeneous medium or interfaces between two media 33, respectively. In this study, we introduced a supercontinuum and super-wide-tuning integrated multimodal platform for label-free evaluation (SIMPLE), which was achieved by combining confocal fluorescence microscopy (CFM), simultaneous label-free autofluorescence-multiharmonic microscopy (SLAM), autofluorescence lifetime imaging microscopy and a chain of autoregulations (Figure 1). The pathological and metabolic analysis of metastatic colonization were performed successively and effortlessly by single- and multiphoton excitation and intensity and lifetime acquisition, featuring fast, large-field and subcellular-scale imaging of exogenous and endogenous fluorophores and nonlinear coherent scattering.

The first module of the SIMPLE is CFM, which is composed of a multicolor CW laser with a corresponding descanned detection unit for initial histopathological examination. The second module employs an automated ultrafast tunable laser, characterized by wide tuning from 700 nm to 1300 nm and pre-compensation of dispersion resulting from optical component delivery, to enable NIR I and II excitation for selecting optimal multiphoton autofluorescence and multiharmonic generation (MAMG) acquisition. However, the broadband tuning would inescapably require complicated and tedious collimation; hence, an AOM and an autoalignment system (AAS), housed in the incident optical unit for the optimization of the IR laser path, is incorporated to modulate the laser beam and stabilize the laser power rapidly (within 3s). Four nondescanned detectors (NDDs) along with two descanned detectors were used to collect three-photon autofluorescence (3PA) NADH, 3PA FAD, two-photon autofluorescence (2PA) FAD, SHG and THG signals, enabling label-free cancer diagnosis, rapid metastatic-margin assessment and effective pathological-feature identification. The third module is based on the descanned light path and the time-correlated single-photon counting (TCSPC) components (SPC-150, SPC-830 and dual DCC-100, Becker & Hickl GmbH) to link between a supercontinuum (400-2500 nm) picosecond laser and two high-speed time-resolved detectors. Combined with the phasor approach, it can further obtain the fluorescence lifetime and ratio of free and bound NADH (FAD) for metabolic analysis. The multicolor CW laser, the red-NIR femtosecond laser, the supercontinuum picosecond laser and the total 8 detection channels in the SIMPLE system can be tailored to various needs with the assistance of the primary DM (a fast interchangeable filter wheel that separates the exciting VIS and NIR lights and reflects the emission signals) and the secondary DM (that shifts the descanned detection to the NDD unit).

## Results

### Whole-colony metastatic analysis by SLAM

SLAM offers an approach for the rapid, stain-free histopathology of fresh tissue samples, rather than conventional histopathology with lengthy turnaround times for tissue fixation, processing and staining 15. To evaluate its capabilities in studying the tumor microenvironment, we used the high-mortality pancreatic cancer mouse model 34 and surgically removed the advanced tumors and liver tissues one month after *in situ* carcinogen injection. Before nonlinear analysis of metastatic colonization, CFM was employed initially with the help of different histological examinations (Note S1) to distinguish different tissues/regions of pancreatic cancer (Figure S2A) and the corresponding liver metastasis (Figure S2B).

**Figure 2:**
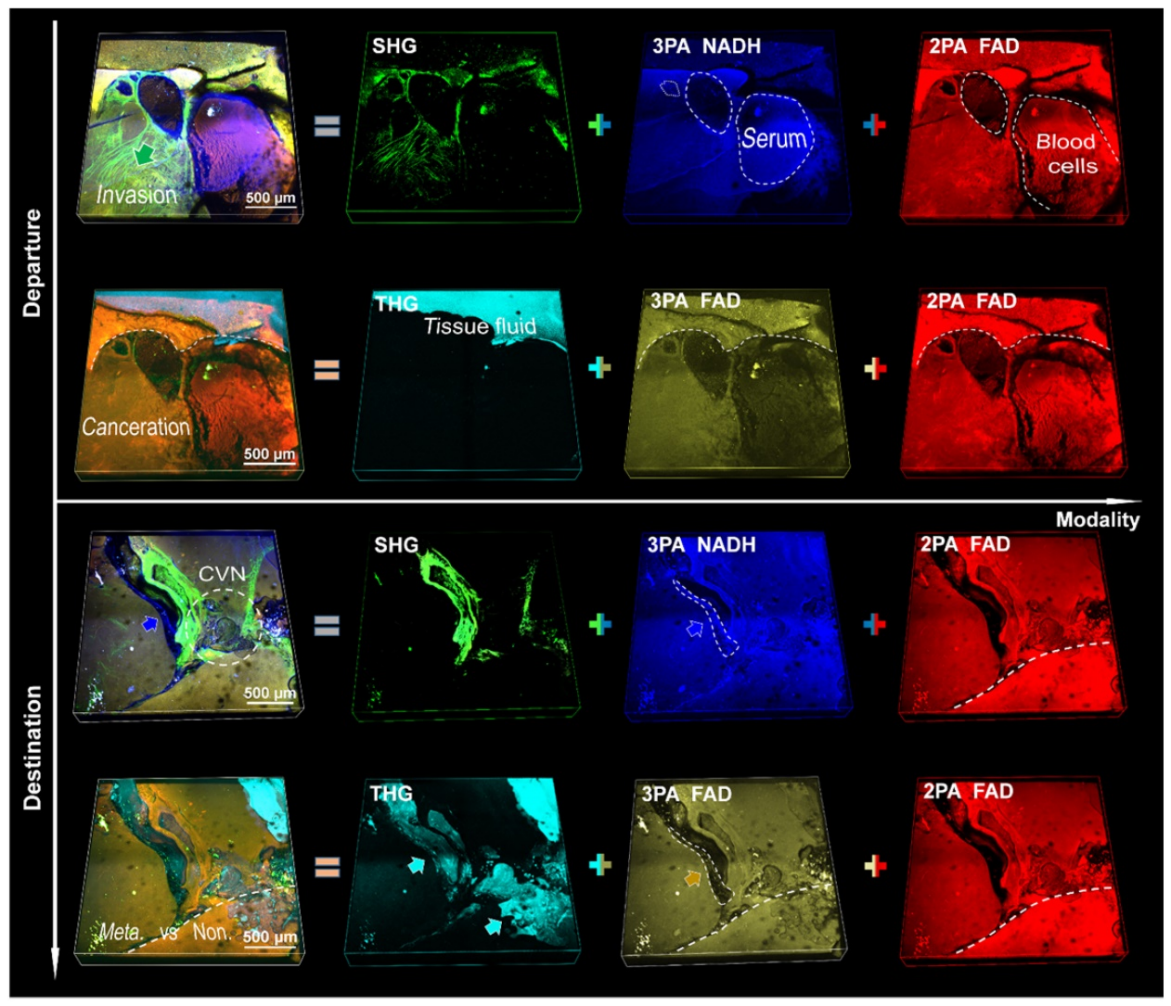
3D SLAM images (2.36 × 2.36 × 0.33 mm^3^) of fresh liver tissues from metastatic pancreatic cancer. The upper two rows are a departure station of invasive cells (a potential inlet of hematogenous spread), while the bottom two are a destination approximately 5 mm away from the departure station. First column: superposition of individual channels in the same row. Second column: angiogenesis-accommodating collagen reorganization revealed by SHG and tissue fluid indicated by THG (cyan arrows). Third and fourth columns: metastatic optical signatures by partially colocalized 3PA NADH, 3PA FAD and 2PA FAD signals. Dashed circles in the first column: collagen and vesicle network, in the third column: 3PA-NADH-visible serotonin, in the last column: FAD-differentiable erythrocytes. Dashed line: discernible margin of cancerization. Blue and yellow arrows: 3PA-visible cancer-associated stroma (vesicles).

**Figure 3:**
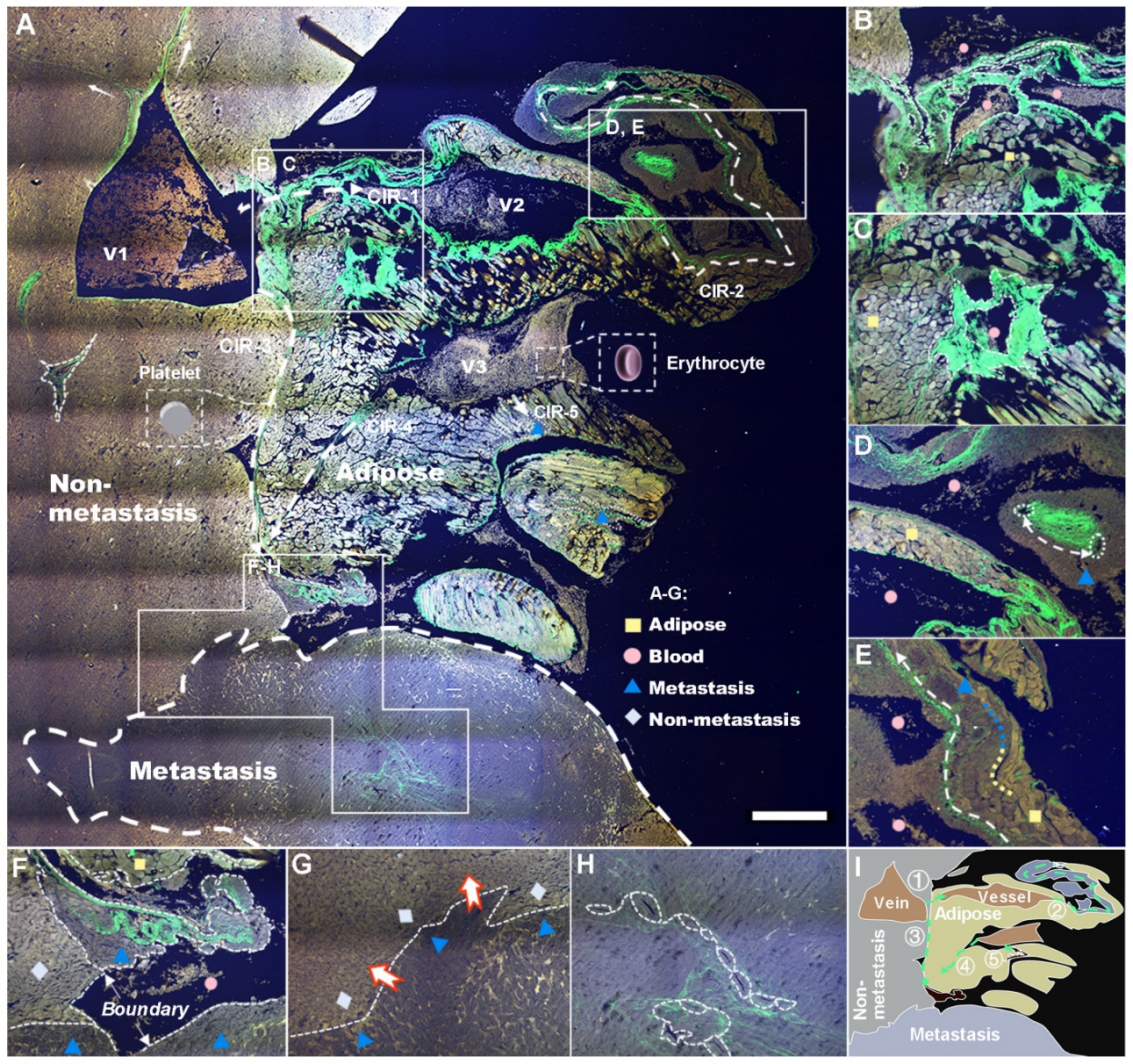
Label-free whole-colony imaging of liver section of metastatic cancer by SLAM. Pseudocolor presentations of blue, green and red were merged by the selected 3PA NADH, SHG and 2PA FAD channels at 1140 nm excitation, respectively. **A,** Full view of the metastatic colony with an area of 5.4 mm × 5.4 mm (5 μm deeper than Figure S2B). White squares: regions of interest magnified in B-H, white dashed arrows: CIRs-1-5, pink triangle: intravascular inflammation 41. **B,** SHG-localized tumor neovasculature (dashed lines) developing on CIR-1. **C,** A strong SHG signal from irregular vessels surrounded by horizontal and vertical lipids. **D,** CAC-bridging (double-head arrow) vessels on CIR-2 implying the material or information exchange of CAVs. **E,** Dysregulation of lipolysis (blue-yellow line) to support the uncontrolled tumor growth and invasion (white dashed arrow). **F,** A junction of adipocytes, erythrocytes, hepatocytes, cancer cells and collagen fibers at the end of CIRs-3 and 4. **G,** Battlefield of cancer infiltration (red-edge arrows) into the normal region. **H,** The concentrated CAVs forming vasculature within a square millimeter (Figure 3H). **I,** A minimal illustration of the major regions and invasion routes. Scale bar: 500 μm.

We applied a pre-compensation for group delay dispersion (GDD) of 7,000 fs^2^ to achieve a low power (<40 mW at 1140 nm) excitation of MAMG. By contrast, the average power to excite collagenous fiber was typically 200 mW at 800 nm 35. Discussion of the focal shift and estimation of the optical resolution of the system could be found in the methods. Our results show that there was no 3PA or THG signal under NIR I excitation, the THG signal under shortwave NIR II excitation was weak and indistinct, and no 3PA NADH signal was visible at 1300 nm excitation (see contradistinctive imaging by 800 nm, 1080 nm and 1300 nm in Figures S3-5, respectively; Figures S3 also serves as an aid to the following analysis). Therefore, we selected an excitation wavelength of 1140 nm for maximum gain of metastatic information. Multiphoton signals from NADH and FAD (participating in glycolysis and oxidative phosphorylation in cellular respiration and ATP synthesis, respectively 36) and multiharmonic signals from collagen fibrils and optical heterogeneity are compared in Figures S6 and S7 (unstained sections) and in Figure 2 (fresh tissue specimens). We could collect valuable optical signatures from these images, such as SHG-labeled landing and cancer invasion routes (CIRs) from hematogenous spread, and the crosslinked collagen formed a network to assist cancer invasion inside the nonpalpable tumor. After the aggressive cancer found adaptive circumstances in which to settle down (the bottom two rows of Figure 2), the collagen and vesicle network (CVN) generated during the process of angiogenesis (see details in Figures S6 and S7) would address the needs for sustenance in the form of nutrients and oxygen 19, 37. It is difficult for the THG signal with the shortest wavelength to reach the surface of a thick tissue due to the limited penetration depth of UV light (300 nm-400 nm) through the tissues (in contrast to tissue slices, whose thickness is usually several micrometers); hence, only strong signals from tissue fluid are detectable. The NADH (blue) and FAD (yellow and red) signals are similarly associated with hepatocytes and cancer cells, but the leaky 3PA-NADH signal revealed more ingredients, such as the vascular endothelial cells and serum. We divide the five signals into two groups accordingly: the first (third) row versus the second (fourth) row (Figure 2), which shows that SHG has a higher capability of labeling invasions than THG 17, and no major difference can be found between 3PA FAD and 2PA FAD (since identical substance excited by one- two- or three-photon resulting in the same fluorescence imaging 38). The reason why the 3PA FAD images were given is not only due to its inherent existence between 3PA NADH and SHG, but also to its fifth-order nonlinear process as the 3PA NADH which is beneficial for redox-ratio calculation using fluorescence intensity 17. In addition, when tuning the excitation wavelength from 1080 nm to 1300 nm, the 3PA NADH signal would become sightless and its channel would be substituted completely by 3PA FAD (see Figures S4 and S5), thus, it is benefic to differ 3PA FAD from 3PA NADH beforehand to prevent from signal confusion. However, since the strong 2PA FAD signal of hematopoiesis was superior to its weak three-photon process in identifying cancerous features, and 3PA NADH and SHG could give more angiogenic information, we chose the first group, which are consistently crucial for many cancer diagnoses 15, 16, 39, for a further large-field illustration of pancreatic cancer (Figure S8) and analysis of liver metastasis (Figure 3).

Global pathological analysis achieved by a large-field autofocus algorithm was performed subsequently to test the capability of our SLAM in identifying characteristics of metastatic colonization, and the results are shown in Figure 3A. Four major tissue areas distinguished by multiphoton signals, five probable CIRs labeled by harmonic signals (white dashed arrows 1-5), and three veins along with dozens of cancer-associated vessels (CAVs) can be observed from the situation as a whole. The stacked cancer-associated collagen (CAC) signatures on the right side of V1 indicate a successful CIR-1 into cancer-associated adipocytes (CAAs). The abundant availability of lipids and dysregulation of lipolysis increase the survival of cancer cells, allowing incipient tumors to generate high-grade, life-threatening malignancies (Figure S3A) by angiogenesis 40. V2 and V3, characterized by stronger 3PA-NADH than V1, are affirmatory pathological veins formed as a result of tumor neovascularization (verified further by FLIM). A number of other signatures revealed by the system are illustrated in Figure 3B-H and Figures S3B-I, and we diagram the distinguishable four regions and five routes in summary in Figure 3I. These characterization results demonstrate the capability of SLAM for label-free pathologic diagnosis despite a lack of metabolic analysis. Hence, we next investigated the cellular metabolism of cancer metastasis using FLIM.

### Whole-colony metabolic analysis by FLIM

**Figure 4:**
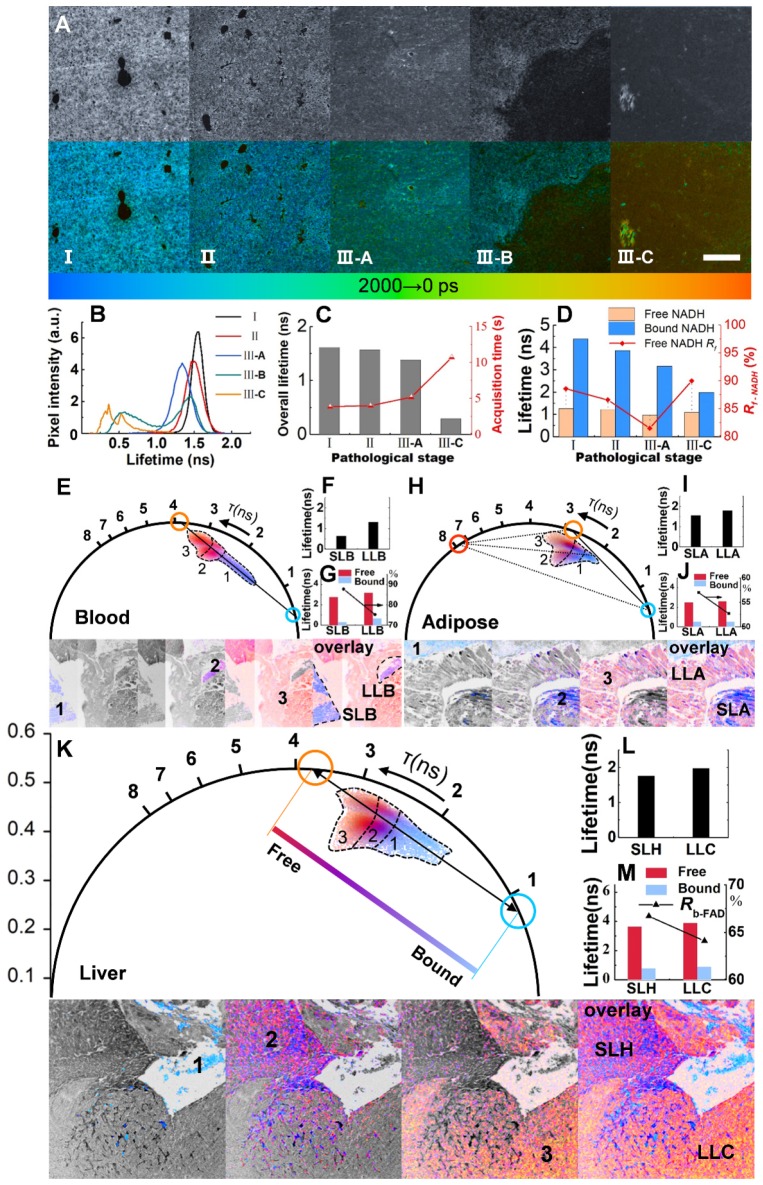
FLIM and phasor analysis of energy metabolism in liver metastatic sections. **A,** Grayscale (accumulated photon counts) and pseudocolor (lifetime) images of different metastatic stages: I: Control; II: Liver with *in situ* pancreatic cancer; III-A: Hepatocytes near metastatic pancreatic cancer; III-B: Border between nonmetastatic tissue and metastasis; III-C: metastatic pancreatic cancer. **B**-**D,** Metabolic features revealed by NADH lifetimes and component ratios (*R_f-NADH_*: ratio of free NADH). **E**-**K,** Phasor analysis (with different pseudo colors) of blood, fat, hepatic and cancer cells of metastatic pancreatic cancer based on FAD FLIM data. Blue (short lifetime) and orange (long-lifetime) symbols represent the bicomponent decomposition (E, K), and another cursor in red (H) indicates the third component. The cluster areas (1, 2 and 3) correspond to different lifetime species in the pseudocolor images below. **F**, **I** and **L**: Overall lifetimes. **G**, **J** and **M**: Lifetimes and ratios (*R_b-FAD_*) of the components. Scale bar: 200 μm.

Lifetime analysis, which is sorely lacking in many multimodal studies 15, 17, is essential for separating between free and protein-bound NAD(P)H and FAD 42-44. Together with the phasor approach 24, 45, lifetime analysis can be a complementary tool for the identification of metastatic specificity. Shifting the 2nd DM to the descanned optical path, tuning the supercontinuum picosecond laser to 400 nm and expanding the pathological tissues to five stages, we tested the discrimination of the FLIM module as shown in Figures. 4A-D (the average power at the sample plane was approximately ~10 nW; note that this is the single-photon case, and we can see Note S2 for two-photon homophyly). The lifetimes of different stages (Figure 4A) shifted peaks (Figure 4B) and downward changes (Figure 4C), as well as the component lifetimes and fractions (Figure 4d,* p* < 0.0001, see more in Note S3). However, there have been a large number of reports on the variations in metabolic pathways revealed by NADH 36, 46, 47, and there is still a need for more discussions on FAD. Moreover, instead of aiming primarily to determine lifetimes or decay components by FLIM, we adopted a phasor approach to separate the direct fluorophore fraction of free and bound FAD (phase analysis of NADH generate a figure similar to that of FAD).

We next changed the excitation wavelength to 450 nm and applied a couple of filters as longpass (LP) 500 nm and BP 550/40 for FAD. Shifting from a longer lifetime in the free state to a shorter lifetime in the bound state, FAD shows a reverse trend compared to NADH in many ways (Note S3). The blood areas in liver metastasis are analyzed first due to the quasi-linear distribution in the phasor plot (Figure 4E). The decomposition of the bicomponent is represented by a blue cursor (short lifetime) and an orange cursor (long lifetime). The line connecting these two cursors shows the fractions of autofluorescence arising from its free/bound form and hence is called the metabolic trajectory 42, 45. The vessels in the colony can be separated into two species, short-lifetime blood (SLB) and long-lifetime blood (LLB), according to their different locations close to either the bound or free state. A shift approaching the free state along the trajectory indicates the metabolic change in erythrocytes from oxidative phosphorylation to glycolysis. The separate clusters 1, 2 and 3 in Figure 4E correspond to SLB, LLB and cancer areas displayed in the below lifetime-colored images. The average fluorescence lifetime (*τ_m_*) shifts from 0.63 ns for SLB to 1.31 ns for LLB (Figure 4F) with a component lifetime increment of ~ 0.4 ns (Figure 4G). The ratio of bound FAD, *R_b-FAD_* (calculated by the equations described in Note S4), decreases by approximately 12.5% (Figure 4G), suggesting a reduction in aerobic respiration. Adipose tissue, due to the triangle-like phasor distribution, is decomposed by the tri-component (Figure 4H). In addition to short-lifetime adipose (SLA) and long-lifetime adipose (LLA), a much longer lifetime species, of which the origin is still unknown but related to lipid oxidation, has been observed to have a lifetime of ~7.8 ns 42, 45. The *τ_m_* increases from 1.56 ns for SLA to 1.79 ns for LLA (Figure 4I) with a decrease in *R_b-FAD_* from 57.0% to 52.8% (Figure 4J). The shifts toward high *τ_m_* and low *R_b-FAD_* reveal a change to glycolysis and, possibly, to cancerous behavior. The fraction of the third lifetime species is calculated to be no more than 5%. For liver tissue, without the lipid oxidation product, the phasor distribution is also analyzed by bi-component decomposition (Figure 4K). As expected, metastatic cancer shows essential variation in fluorescence lifetime due to its metabolic heterogeneity. LLB, short lifetime hepatocytes (SLH) and long-lifetime cancer (LLC) can be differentiated by their locations on the trajectory. It is calculated that *τ_m_* and *R_b-FAD_* are 1.76 ns and 66.7% for SLH and 1.98 ns and 64.1% for LLC (Figures. 4I and M). These metabolic features demonstrate that changes in metastatic colonization can be probed by the characteristic parameters of free and bound FAD, which forms the basis for the analysis of large-field FLIM below.

**Figure 5:**
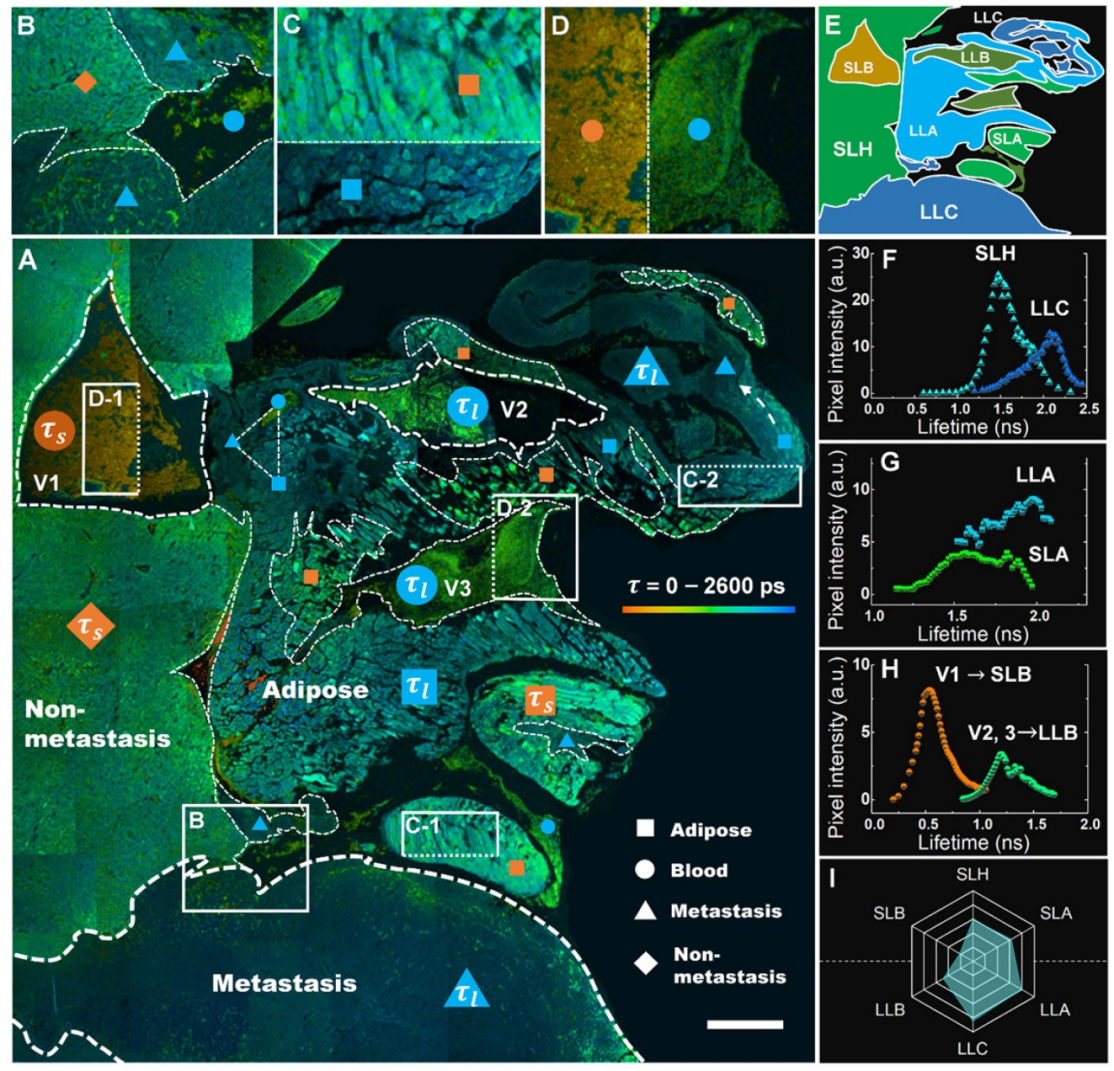
Metabolic features of liver section of metastatic colonization revealed by FLIM. **A,** Large-field imaging of a metastatic colony (the same area as Figure 3A). The fill colors of orange and blue in the symbols (triangle, square, rhombus and circle) correspond to the short lifetime *τ_s_* and long lifetime *τ_l_*. **B-D,** Contrast images of SLH and LLC (B), SLA (C-1) and LLA (C-2), and SLB (D-1) and LLB (D-2). **E,** A minimap simply illustrating the distribution of the six species.** F-H,** correspond to the *τ_m_* histograms of SLH and LLC, SLA and LLA, SLB and LLB, which are aggregated by the whole area of A. **I,** A hexagonal radar lifetime profile of the six species (averaging F-H): the upper three are shorter lifetime components, while the lower three are correspondingly longer ones. Scale bar: 500 μm.

A comprehensive analysis of the lifetime distribution of endogenous fluorophores is shown in Figure 5, which provides whole-area nondestructive probes for the critical free/bound state of intracellular FAD metabolism in the tumor microenvironment. A number of cancer-associated optical signatures offered by Figure 3 can be found likewise in Figure 5A, e.g., tens of CAVs caused by angiogenesis clustering in LLA and LLC (dashed triangle) and dysregulation of lipolysis in LLA supporting the local invasion of LLC (dashed white arrow). Moreover, as expected, liver metastasis displayed essential decay variations due to metabolic heterogeneity. Figure 5A shows that the *τ_m_* values of hepatocytes, adipocytes and erythrocytes increase with the infiltration of cancer; their lifetime images are given specifically in Figures. 5B-D, demonstrating that each group can be separated into antitheses as mentioned above. Therefore, combining the nonlinear MAMG and phasor-lifetime, we can further divide the whole colony into six regions: SLH, LLC, SLA, LLA, SLB and LLB, as illustrated in Figure 5E. Figure 5f shows that the fluorescence lifetimes of SLH and LLC aggregated by the entire Figure 5A are 1.2-2.0 ns and 1.5-2.3 ns, respectively, with a 0.6 ns peak gap. The lifetimes of SLA and LLA (Figure 5G) vary differently in the ranges of 1.3-1.9 ns and 1.5-2.2 ns, with a large overlay resulting from wide LLA-LLC entanglement. The average lifetimes of LLB (Figure 5H) can be used for distinguishing CAVs from normal vessels; for instance, in addition to the morphological and strong-NADH-signal characterizations provided by Figure 3A, V2 and V3 are demonstrated to be CAVs because their lifetimes are twice that of V1. A hexagonal radar of the six lifetime species (Figure 5I) shows that the overall lifetimes of blood, adipose, liver and cancer have the following relationship: SLB < LLB < SLH < SLA < LLA < LLC (*p* < 0.0000).

## Conclusions

It is well known that treatments for metastatic cancer are minimally effective. Pancreatic cancer currently has the lowest 5-year relative survival rate 5; to increase survival, it is essential to improve our strategies to identify and study it and its metastasis precisely and comprehensively 8. The SIMPLE system we developed, which is based on various automated tunable lasers, autoadjustment components and a total of eight detection channels to cover different functions for different needs, offers the possibility. The nonlinear analysis carried out in the NIR I and II windows with two descanned detectors and four NDDs ensured the fast and maximum gain of pathological information and ultimate capture of significant relationships between metastases and non-metastatic tissue. The FLIM analysis combined with the phasor approach, in spite of longer time to acquire data (<10 s for normal tissue and >15 s for tumor tissue at 256 × 256 pixels), demonstrated the short and long lifetime deviation of aerobic and anaerobic energy metabolism, which, together with the SLAM technique, further differentiated metastasis-related and unrelated features specifically (Figure S13). Without the diverse modalities of SLAM, the short-wavelength linear system cannot achieve fresh tissue imaging (by NIR) and CIRs analysis (by SHG); while without the phasor-FLIM contrast, bias along the metabolic trajectory indicating the change between oxidative phosphorylation and glycolysis is absent. These multi-dimensional messages are usually unavailable on an individual traditional system, and inconveniently obtained by multiple separate systems which require sample transfer and FOV re-finding. With such an integrated multi-dimensional system, we can search the metastatic region by 3PA NADH and 2PA FAD imaging, analyze the cancer invasion by SHG signal, which, can be achieved simultaneously with the multiphoton signals, and then reveal the affected cellular metabolism by phasor-FLIM. It is possible to obtain all this information in the same area of the same sample in a short time. This synergistic effect demonstrates the sufficient capability of the SIMPLE system to achieve label-free whole-colony visualization and cellular-scale analysis of CIRs and metabolism, and enable the evaluation of the pathological features of metastatic colonization by a noninvasive method that requires less preparation, and provides more information. The optical signatures collected by the SIMPLE system can be used in the construction of an optical database and further study of the dynamic mechanisms and biochemical processes of metastatic cancers. Nevertheless, future investigations will be focused on integrating fiber-bundle endoscopy 48, 49 and deep-learning algorithm 50 into the system to provide a significant boost to multimodal optical imaging and all-optical label-free disease diagnosis.

## Methods

### Sample preparation

Male 6-week-old mice (c57BL6) were purchased from Guangdong Medical Laboratory Animal Center. The mice were housed in the animal facility of the Photoelectric Institute of Shenzhen University. All animal procedures were approved by the Laboratory and Equipment Department and Institutional Ethical Committee of Animal Experimentation of Health Science Center, Shenzhen University. Murine pancreas carcinomas were *in situ* induced by the pancreas implantation of Panc02-H7 cells (5*105 cell 50 ul^-1^), which are more aggressive than other sublines of Panc02 34, into the pancreas of 6-week-old male mice (c57BL6) via open surgical technique. Mice had a laparotomy one month after tumor cell inoculation when the pancreatic cancers were terminal. After surgical removal, the pancreas and liver (with or without metastasis) were dissected into small blocks, some of which were placed in saline for 3D nonlinear imaging, some were cut into a series of 5-μm sections for unstained (SLAM and FLIM) and stained (histology) sectional applications using a vibratome, while others were fixed in zinc-buffered formalin (Anatech) and then embedded in low-melting paraffin for reserve. Histological sections with hematoxylin and eosin (H&E) staining, toluidine blue (TB) staining, Van Gieson's (VG) staining, periodic acid-Schiff (PAS) staining (each 5 μm thick and continuous in depth) were obtained to evaluate pathological characterizations.

### Optical setups

The CFM was equipped with a quadruple-output CW laser module (MHF450AA, Nikon) and two of the wavelengths (488 nm and 561 nm) were selected for H&E imaging. To separate different channels, the following combinations of LP dichroic mirrors and BP filters were set up: (1) LP 560 nm and BP 525/50 nm; (2) LP 640 nm and BP 595/50 nm. The hybrid (galvano-resonant) scan head (MHA50200, Nikon) is capable of high-resolution (up to 4096 × 4096 pixels) or high-speed scanning (up to 420 fps). The image size obtained by the 10 × objective (MRD00105, 0.45 NA, Nikon) was 1269.79 µm × 1269.76 µm, 634.88 µm × 634.88 µm by the 20 × objective (MRD70200, 0.75 NA, Nikon), and 122.88 µm × 122.88 µm by the oil-immersion 100 × objective (MRD01901, 1.40 NA, Nikon).

Based on the CFM system, we built the nonlinear imaging module by assembling and synchronizing a femtosecond laser, an incident optical unit, the 1st and 2nd DM and the NDD unit. The excitation light from the automated broadly tunable (700-1300 nm) femtosecond laser (pulse width: 100 fs, repetition rate: 80 MHz, Chameleon Discovery, Coherent) with GDD pre-compensation for optimized average power at the sample plane (40 mW for 1140 nm) was collimated by the AOM-based autoalignment system and directed into the hybrid scanner through the 1st DM (a filter wheel that is interchangeable for 405/488/561/640/IR laser). The motorized secondary DM (with IR-cut filter cube) could rapidly switch the backscattered emission signals to either the two descanned detectors or the four GaAsP NDDs. The light crosstalk autofluorescence and harmonic generation signals were then separated by the combinations of LP and BP filters (Tables S1-3) and purified by mathematical function of ImageJ (National Institutes of Health).

FLIM measurements were carried out by synchronously connecting the supercontinuum (400-2500 nm) picosecond laser (pulse width: 6 ps, repetition rate: 40 MHz, WL-SC-400-4, Fianium), the galvano scanner and two high-speed time-resolved detectors (HPM-100-40, Becker & Hickl GmbH) to the TCSPC modules (SPC-150, SPC-830 and dual DCC-100, Becker & Hickl GmbH). Two laser wavelengths of 400 nm and 450 nm were selected for the excitation of NADH and FAD, respectively. The average powers output from fiber port were ~1 μW for 400 nm and ~2.2 μW for 450 nm, and delivered to the sample plane were ~10 nW (measured by PM130D, Thorlabs). To purify different autofluorescence signals, we applied two combinations of filters: (1) SP 450 nm and BP 450/40 nm for NADH; (2) LP 500 nm and BP 550/40 for FAD.

### Performance evaluation

Determination of optical resolution by fitting Gaussian functions to experimental data is a key issue in image processing 51. We estimated the Rayleigh resolution of the system using 20-nm-diameter fluorescent beads (F8888, Thermofisher) as shown in Figure S14A-C. The resolution for CFM (FLIM) was 461 ± 6 nm (mean ± s.e.m, *n* = 15), approaching the highest optical resolution at 525 nm emission for the 20 × 0.75 NA objective; whereas in SLAM the resolved value reduced to 444 ± 8 nm (n = 15) for 800 nm excitation, 469 ± 12 nm (n = 15) for 1140 nm excitation and 531 ± 13 nm (n = 15) for 1300 nm excitation. For a higher NA =1.40 objective the Rayleigh resolutions (Figure S14C) were measured as 241 ± 4 nm (n = 15) for Confocal, 259 ± 3 nm (n = 15) for 800 nm excitation, 268 ± 3 nm (n = 12) for 1140 nm excitation and 306 ± 3 nm (n = 15) for 1300 nm excitation. The resolution of the system is sufficiently high to reveal collagen fiber orientation of the cancerous zone (Figure S14D-G). Also, the contrasting spectral wavelengths involved in confocal and nonlinear imaging inevitably result in chromatic aberration, which are maximally eliminated by the apochromatic (Plan APO) objectives. However, focal-point shift remains slightly between distant wavelengths which were determined by a nanopositioning systems as (z = 0 for CFM 488 nm excitation): ∆z = 2.0 μm for 800 nm excitation, ∆z = 11.4 μm for 1140 nm excitation and ∆z = 16.7 μm for 1300 nm excitation.

In addition, although a sufficiently compressed pulse (e.g. 5 fs FWHM) with a Gaussian profile durations produced by the mode-locking system seems the best, its corresponding spectral bandwidth (Fourier transform) is unnecessarily 200 nm at 800 nm 52. Adapting the laser bandwidth (e.g. 10 nm) to match the two-photon absorption spectrum is inevitably related to the increased pulse duration (e.g. 100 fs). These, as a result, are the parameters of our systems (the pulse widths of the femtosecond laser measured by autocorrelation profiles were 126 fs at 800 nm, 107 fs at 1080 nm and 100 fs at 1140 nm). However, implement of supercontinuum can achieve freedom of bandwidth without affecting the pulse duration and hence is investigated in ultrawide band femto- and picosecond lasers 53. We carried out more imaging of liver slice and thick-tissue using our supercontinuum picosecond source as shown in Figure S15. The results demonstrated (1) lower intensity, more blur and noise of slices imaged by picosecond laser as compared to that of femtosecond laser and (2) lower SNR of the thick tissue image by FLIM (Figures S15C and D) than that of the slice images (Figures 4 and 5), with no higher imaging speed than that of SLAM. Even more, to achieve higher SNR an excitation power of more than 60 mW (800 nm) at the sample plane was required, which predictably leaded to photodamadge. Therefore, the broadly tunable femtosecond laser is indispensable for this SIMPLE system.

### Image processing

The SLAM images were obtained by averaging four frames and adjusted by look up table (LUTs). The THG and 3PA signals had a low SNR and were therefore further regulated by tuning the dynamic range (brightness/contrast) in ImageJ to better show their discernible morphological features. The crosstalk of 3PA FAD signals into SHG channels was filtered out by the mathematical function (subtract) in ImageJ. The large-field images were achieved with an aotufocus algorithm (along-z) and a 15% overlay. The 3D images were shown at the maximum intensity projection. The acquired lifetime data, including the photon distribution over the coordinates of the scan and the time during fluorescence decay, were processed by SPCImage (Becker & Hickl GmbH). The histograms of SLH and LLC (Figure 5F), SLA and LLA (Figure 5G), SLB and LLB (Figure 5H) were obtained by separating different tissue constituents via the phasor approach (select cluster) in each image of Figure 5A and then accumulating the pixel counts in each lifetime interval (~30 ps) to form whole-area lifetime histograms by MATLAB.

## Supplementary Material

Supplementary figures and tables.

## Abbreviations

ERK: extracellular regulated protein kinase
MRI: magnetic resonance imaging
CT: computed tomography
PET: positron emission tomography
US: ultrasound
NADH: nicotinamide adenine dinucleotide
FAD: flavin adenine dinucleotide
2PEF: two-photon excited fluorescence
3PEF: three-photon excited fluorescence
SHG: second harmonic generation
THG: third harmonic generation
CARS: coherent anti-Stokes Raman scattering
FLIM: fluorescence lifetime imaging microscopy
SIMPLE: super-wide-tuning integrated multimodal platform for label-free evaluation
CFM: combining confocal fluorescence microscopy
SLAM: simultaneous label-free autofluorescence-multiharmonic microscopy
MAMG: multiphoton autofluorescence and multiharmonic generation
AAS: autoalignment system
NDDs: nondescanned detectors
TCSPC: time-correlated single-photon counting
GDD: group delay dispersion
CIRs: cancer invasion routes
CVN: collagen and vesicle network
CAVs: cancer-associated vessels
CAC: cancer-associated collagen
CAAs: cancer-associated adipocytes
SLB: short-lifetime blood
LLB: long-lifetime blood
SLA: short-lifetime adipose
LLA: long-lifetime adipose
SLH: short lifetime hepatocytes
LLC: long-lifetime cancer
H&E: hematoxylin and eosin
TB: toluidine blue
VG: Van Gieson's
PAS: periodic acid-Schiff
FWHM: full width at half maximum
LUTs: look up table
