# Supplementary material for: Label-free whole-colony imaging and metabolic analysis of metastatic pancreatic cancer by an autoregulating flexible optical system
Source: Theranostics. 2020 Jan 1;10(4):1849–60. doi: 10.7150/thno.40869 (PMC6993220; doi:10.7150/thno.40869)
Supplement: Supplementary file 1 — Supplementary figures and tables. [file thnov10p1849s1.pdf]

Label-free whole-colony imaging and metabolic analysis of metastatic pancreatic cancer by an autoregulating flexible optical system

Binglin Shen†, Junshuai Yan†, Shiqi Wang, Feifan Zhou, Yihua Zhao, Rui Hu, Junle Qu*, Liwei Liu*

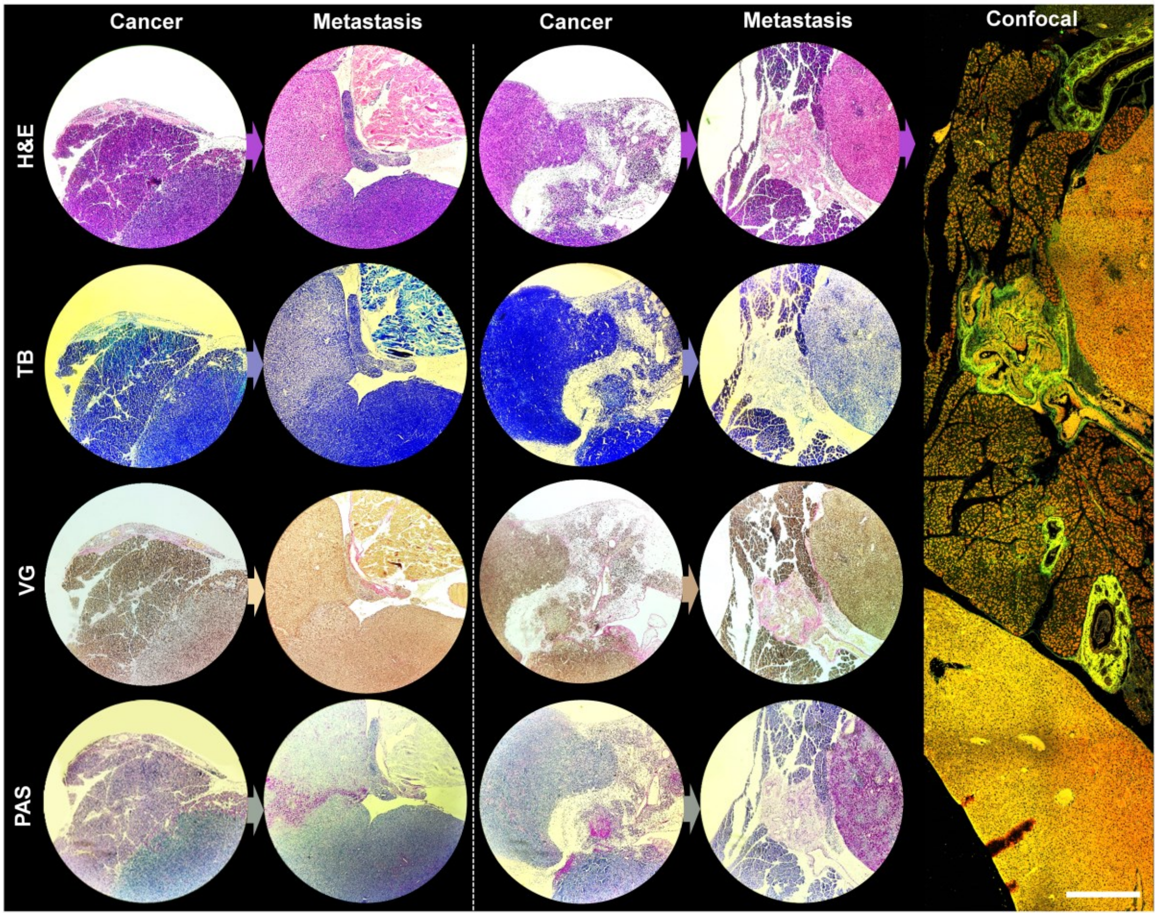

Figure S1. Commercial histological images collected on representative stain sections. Top row: Hematoxylin and eosin (H&E) stains to identify the pancreatic cancers and its corresponding liver metastases; Second row: Toluidine blue (TB) stains to further confirm these cancers; Third row: Van Gieson's (VG) stains to distinguish collagen fibrils; Bottom row: Periodic acid-Schiff (PAS) stains to detect polysaccharides (showing the metabolic activities of the aggressive cancers). Last column: a corresponding confocal large-field image to give the metastatic specification. Scale bar: 500 μm.

Note S1: Primary analysis of the orignal and metastatic cancers by CFM

Before nonlinear and lifetime imaging, we first gave the origin (Figure S2A) and collected sufficient optical signatures (Figure S2B) of liver metastasis by CFM after initial histological examinations (Figure S1) to assist the pathologic analyses of SLAM and FLIM. In Figure S2, we can clearly see the left-side abundant cancer-associated vessels (CAVs) at sites of highly lipolysed adipocytes caused by angiogenesis (Figure S2C) and the middle 1-mm-width irregular vein (Figure S2D) without vascular wall and endothelial cells but many marginal fenestrations making it permeable. After cancer cells succeeded in invading locally through surrounding ECM (extracellular matrix) and stromal cell layers, they intravasated into the lumina of this pathological vein, which is supported by the coexistence of cancer cells and red cells in the inset of Figure S2D. The vein and the CAVs provided possibility of metastasis of the primary cancer to sites distant from homeland. And two major routes of cancer spread are literally being recognized [1, 2]: dissemination via blood vessels and via lymph vessels. After reinitiating the proliferative programs and creating favorable surroundings within the liver, the cancer cells formed a foreign colony as shown in Figure S2B [3]. The abundant available energy stored in lipid and the CAV developments are so important to this hostile colonization that provide plentiful supplies (Figure S2E and further in the next section) [4]. However, the primary function of the CFM is to identify several types of tissue components including adipocytes, collagens, erythrocytes, non-metastatic hepatocytes and metastatic cancer cells, which can be summarized in Figure S2F, for SLAM and FLIM analysis.

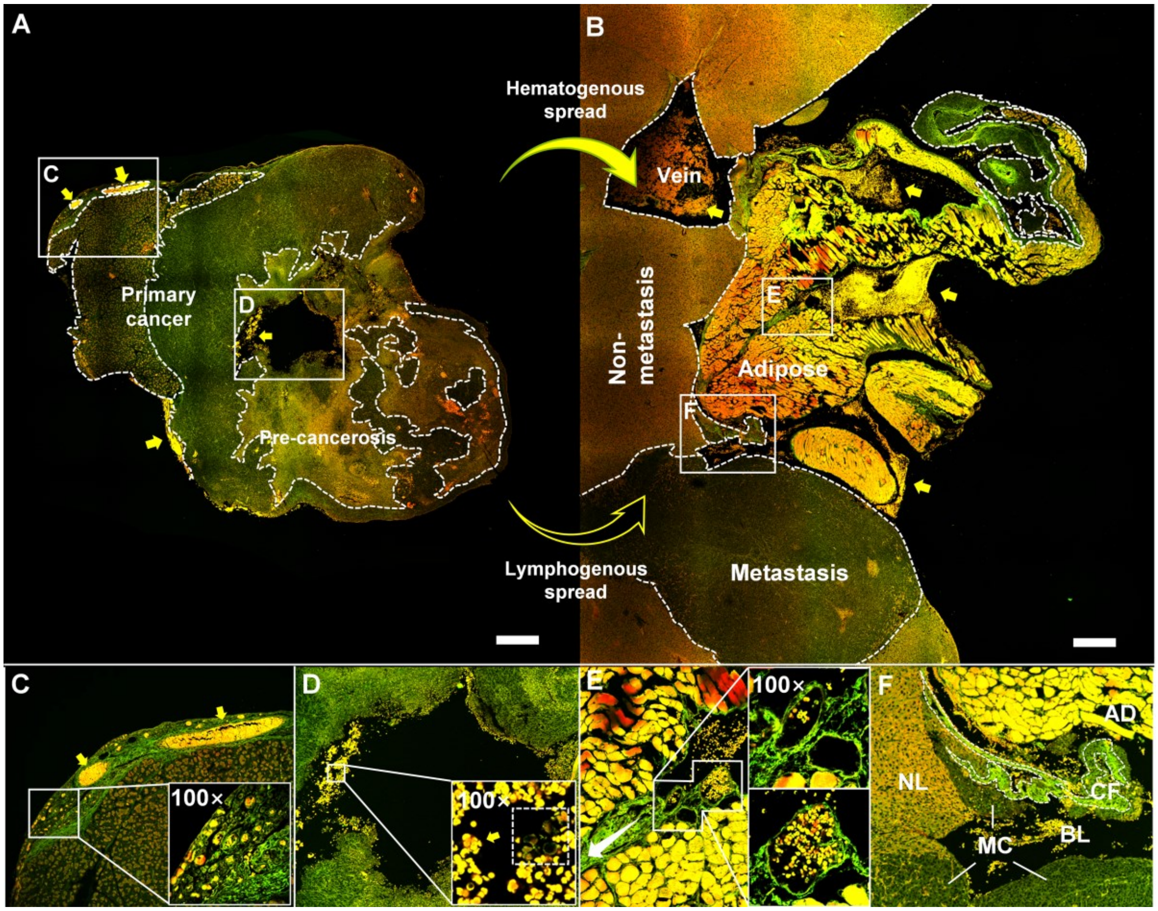

Figure S2 Microenvironment of pancreatic cancer (A) and its liver metastasis (B) by CFM. Pseudo green and red in the image were merged by dual channels of H&E stain simultaneously excited/collected by 488/525 nm and 561/595 nm, respectively. Different regions of interest (white squares) were reimaged emphatically as shown in C, D, E and F. Yellow arrows: erythrocytes; white dashed lines: boundaries; arched arrow: hematogenous spread (yellow-filled) and lymphogenous spread (yellow-edge). **C**, The distinct pathological vessels surrounded by a number of micro CAVs (inset) are adjoining to cancer-associated adipocytes (CAAs). **D**, A 1-mm-width irregular vein in the center of the pancreas. Inset: coexistence of erythrocytes, cancer-associated erythrocytes and cancer cells. **E**, Cancer neovascularization accompanied by cancer-associated collagen. Insets: (Top) The coexistence of perfused and hollow vessels. (Bottom) A mature vessel resulting from angiogenesis. The insets of C, D and E were acquired at 100 × magnification. **F**, A junction of five tissue components. Abbreviation: AD: adipose; BL: blood; CF: collagen fibers (white dashed line); MC: metastatic cancer, NL: non-metastatic liver. Scale bar: 500 μm.

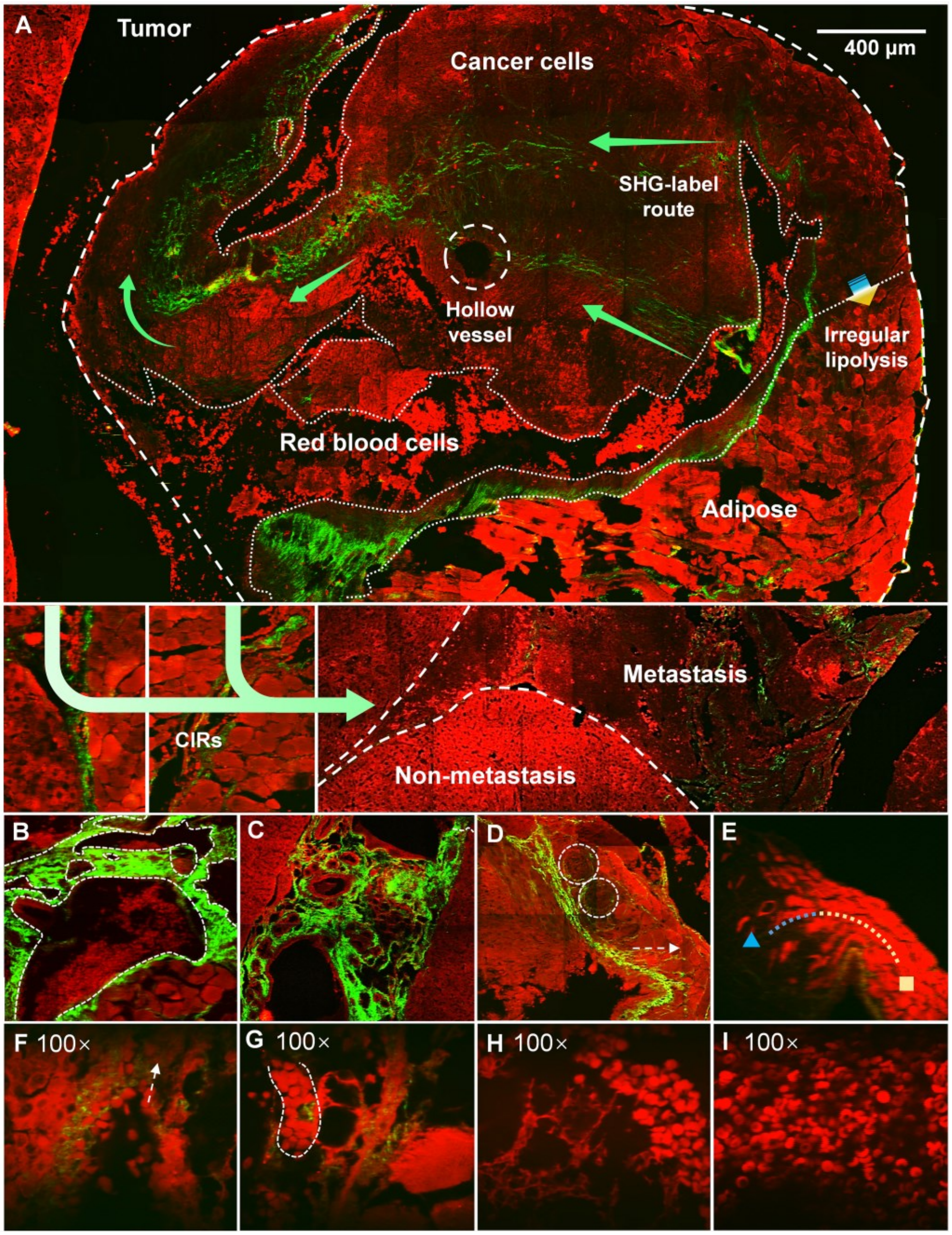

Figure S3 Tumor microenvironment revealed by 2PA FAD (red) and SHG (green) signals at short-wavelength excitation, 800 nm with filters of 400/10 nm (SHG) and 607/70 nm (2PA FAD). **A**, Green arrows: SHG-labeled cancer-invasion routes (CIRs), green-orange arrow: dysregulation of lipolysis offering assistence for metastatic cancer to generate high-grade, life-threatening malignancies by angiogenesis. Second row: local spread through CIRs into the hepatic region and thereby forming a colony. **B** and **C**, Highly tumorigenic neovascularization; **D**, SHG-label infiltration of ECM into adipose (white circles); **E**, The detail of adipocytes either dedifferentiating into pre-adipocytes or being reprogrammed into CAAs which secrete adipokines for stimulating the CIRs. **F**, Intravascular cell recruitment; **G**, A blood clot; **H**, Intertwined fibrins of blood clots; **I**, Non-inflammatory red cells in the same vein.

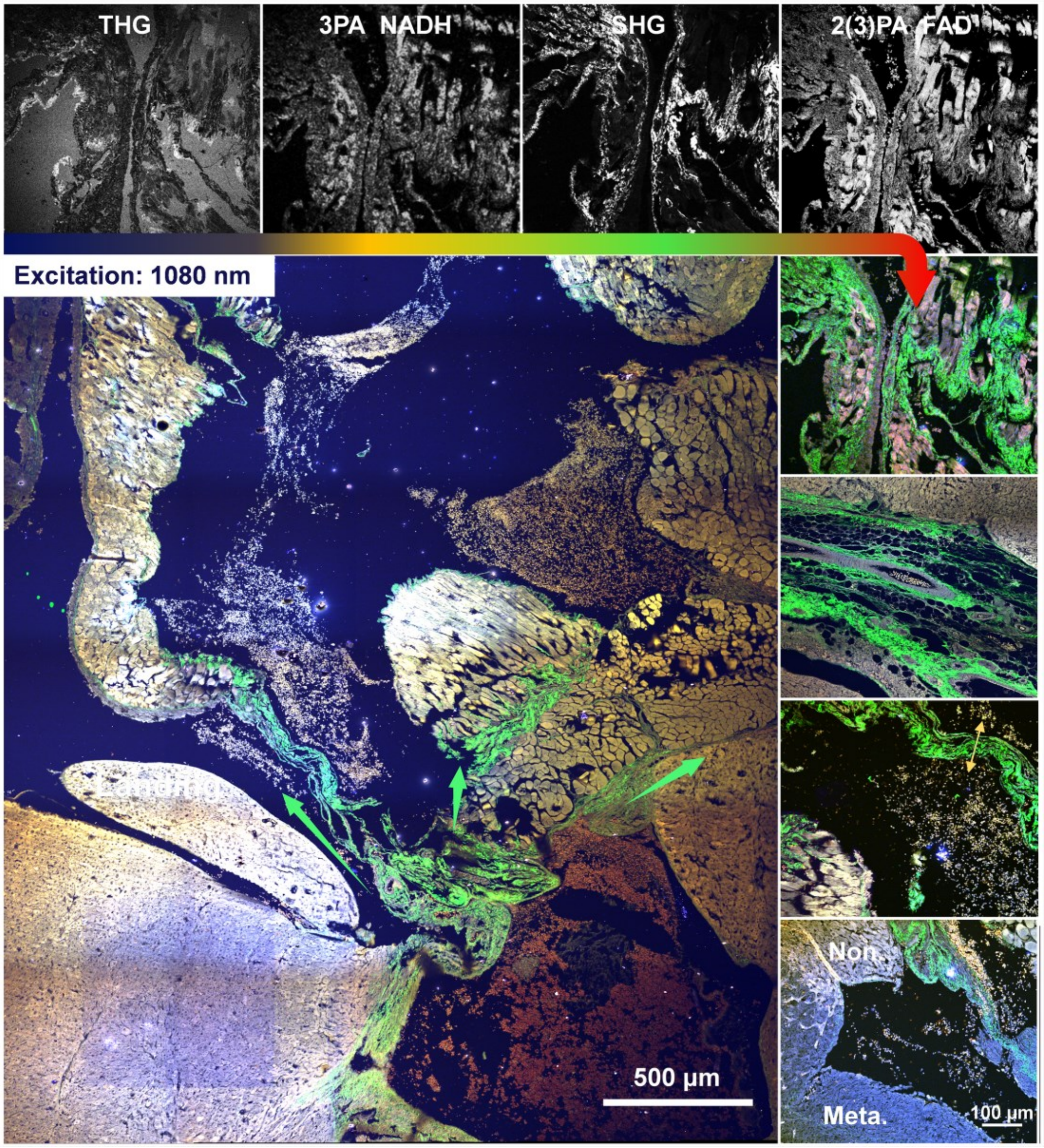

Figure S4. Tumor microenvironment of liver metastasis by SLAM at an excitation wavelength of 1080 nm. The THG, 3PA NADH, SHG and 2PA FAD signals were separated by the specified dichroic mirrors and bandpass filters (Table S1). The THG signals are weak and indistinct due to low transmission (< 40%) of those spike filters (FWHM = 10 nm) with center wavelength around 360 nm. The 1080 nm excitation seems to result in misty, fuzzy and low-SNR THG and 3PA-NADH images and hence "despeckle" is necessarily executed by ImageJ. Further below 1000 nm excitation may result in few collection of THG, and the 3PA NADH could switch to 2PA NADH. However, a majority of cancer-associated characterizations obtained from the left large-field and the right column images, including the SHG-label CIRs, CAVs development, permeable vascular wall (yellow double-head arrow) and non- and metastasis junction, show similarity with 1140 nm excitation.

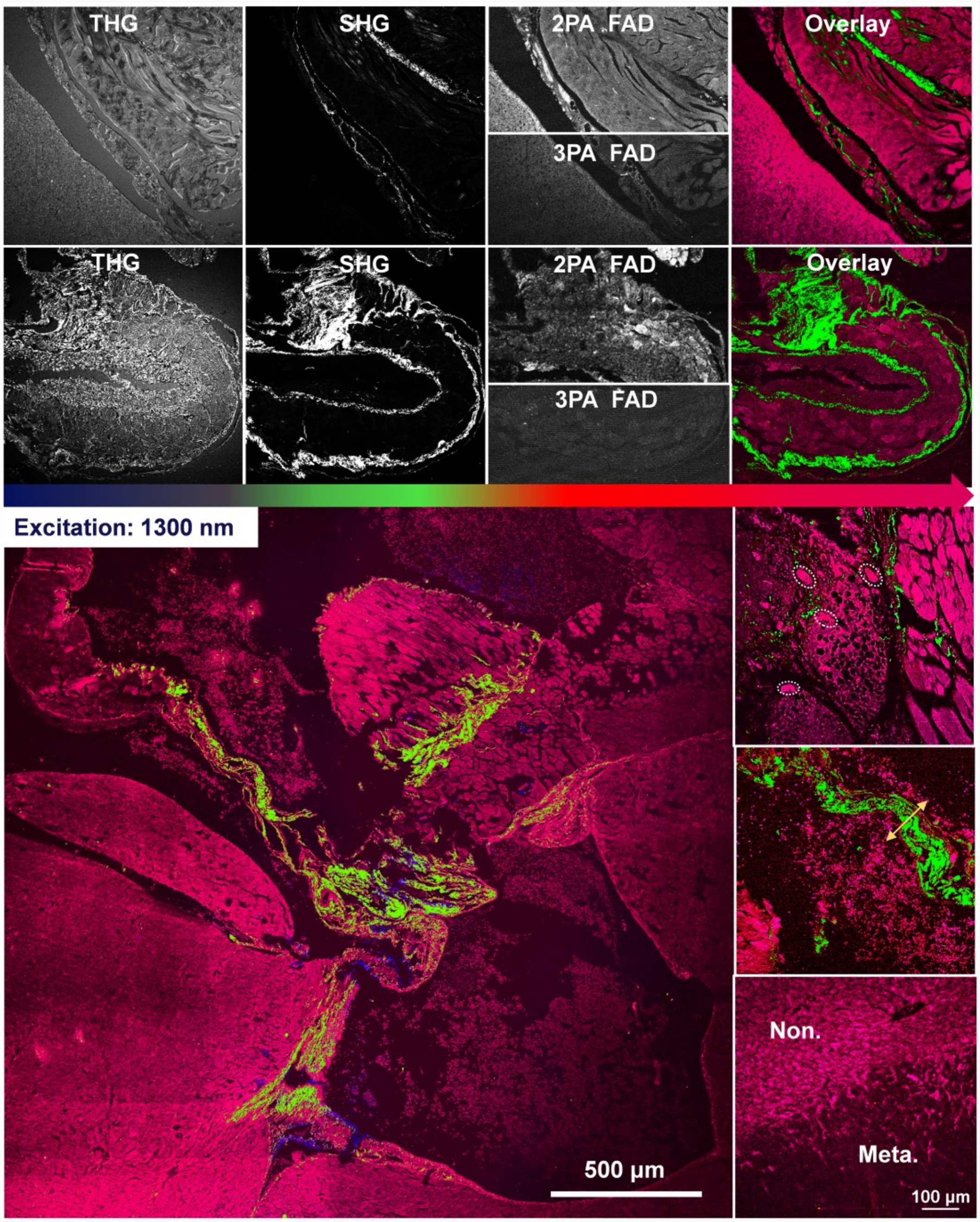

Figure S5. Tumor microenvironment of liver metastasis by SLAM at an excitation wavelength of 1300 nm. The THG, SHG, 3PA FAD and 2PA FAD signals were separated by combinations of dichroic mirrors and bandpass filters given in Table S2. The THG signals are much stronger as compared to 1080 nm excitation. However, the 3PA NADH signal is sightless and its channel can be substituted with 3PA FAD (BP: 550/88 nm). Most of the optical signatures could still be collected, e.g. transformation of adipocytes before (the top row) and after (the second row) the cancer infiltration, CAVs development (dashed circles), permeable vascular wall (yellow double-head arrow) and border of non- and metastasis.

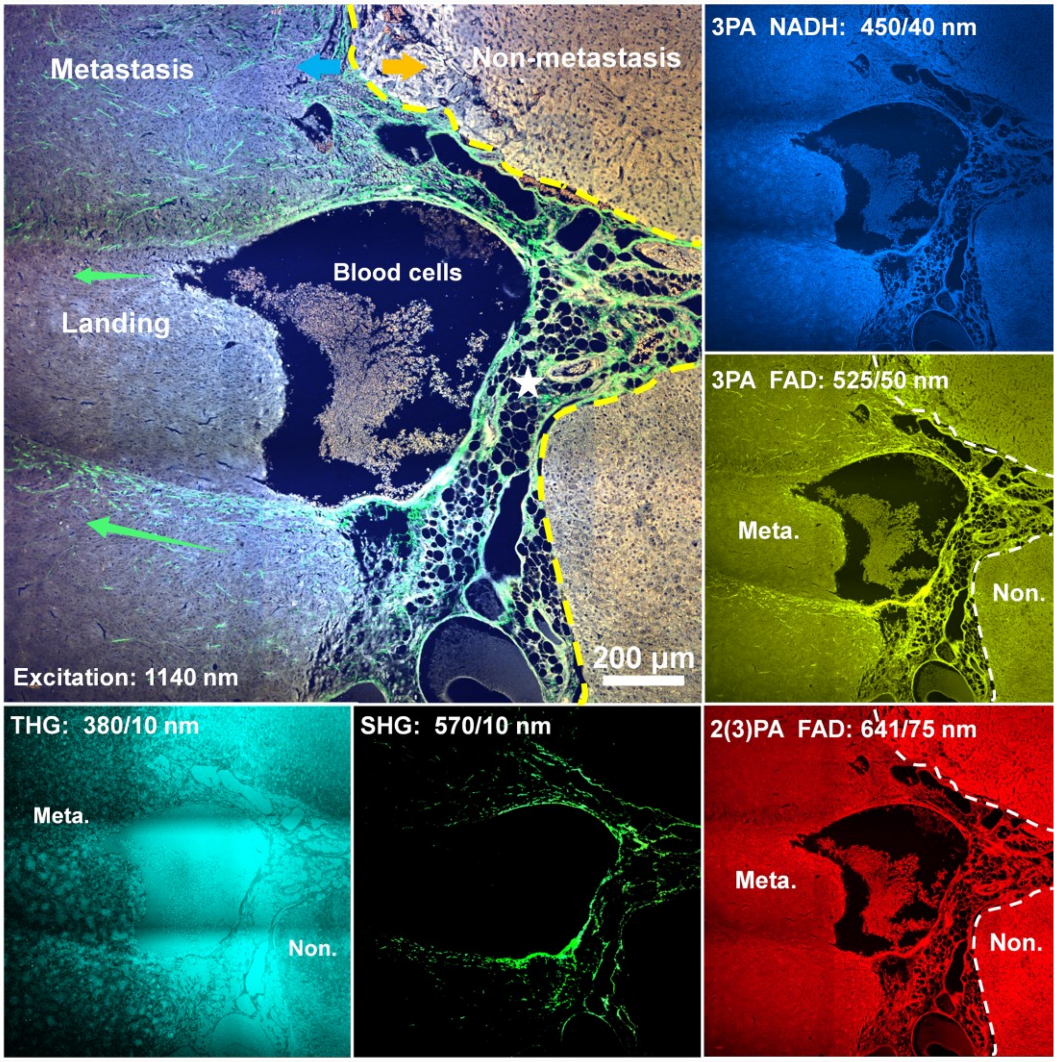

Figure S6. Contradistinctive imaging of 2PA, 3PA, SHG and THG in the mouse liver slice with pancreatic cancer metastasis. According to the excitation wavelength of 1140 nm, befitting filters to purify the optical signals are selected in Table S3. The metastatic areas (periwinkle blue) can be effortlessly distinguished from the non-metastatic tissues. The landing and invasion routes of cancer cells from hematogenous spread, along with aggregation of perfused and hollow CAVs (white star), are characterized by the SHG networks (white star).

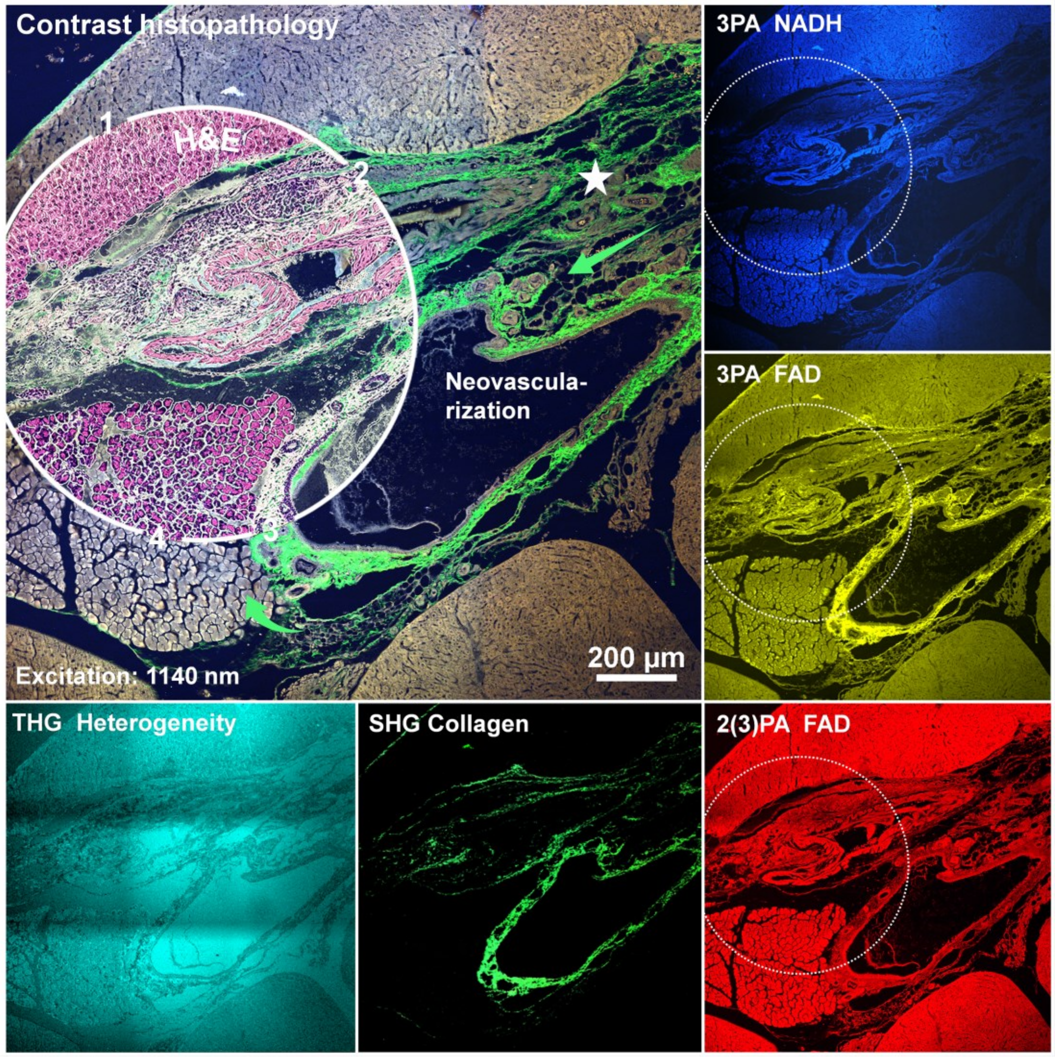

Figure S7. Contradistinctive imaging of SLAM and histopathology in the cancer colony. Hepatocytes (1), cancer cells (2), cancer-associated vessels (3) and adipose (4) are indentified clearly by H&E and SLAM. The dashed circles in the right column indicate the same area of H&E stain, showing the great capability of 2PA and 3PA signals in discrimination of individual specialized cell types. Optical heterogeneities like interfaces between different media are recognized by THG, but it seems not be able to distinguish cell specificity; Whereas SHG networks (white star) still powerfully characterize the metastatic neovascularization.

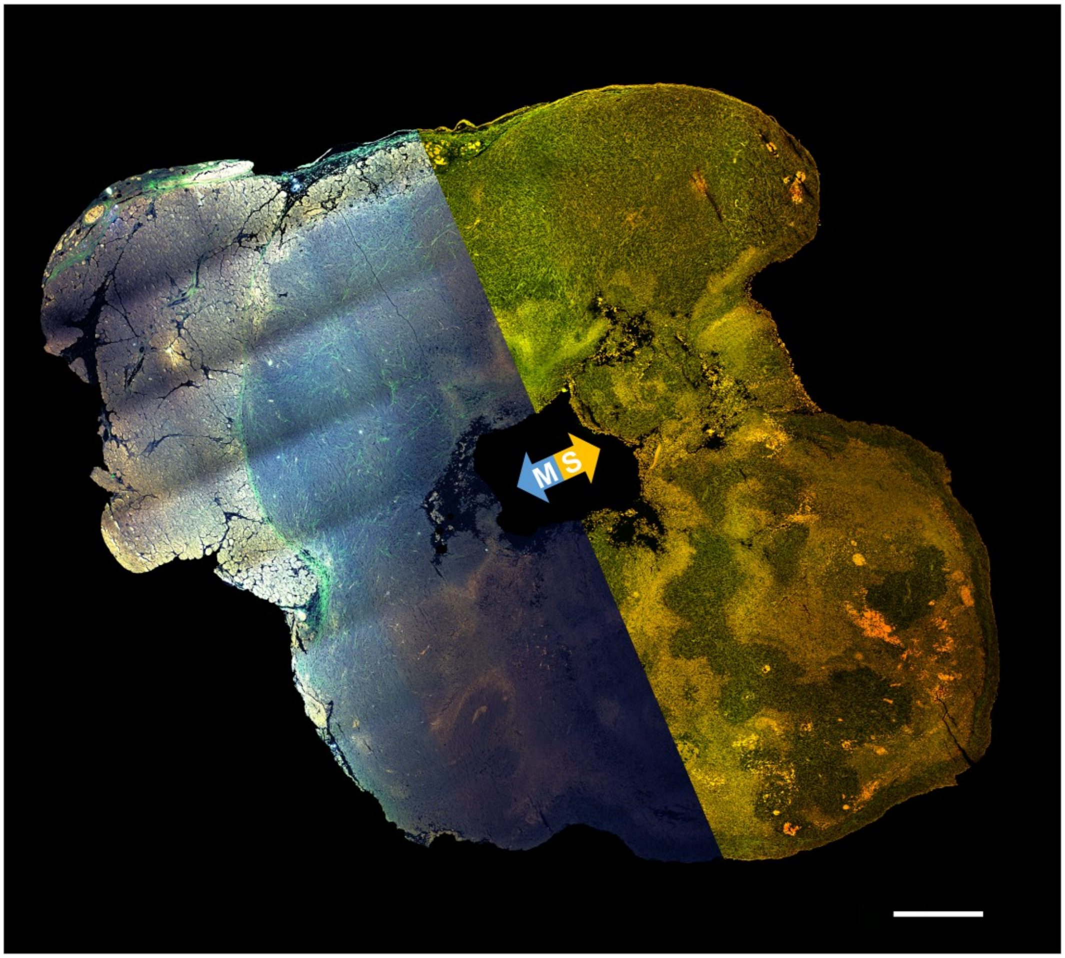

Figure S8. Comparison of the whole pancreatic cancer between label-free SLAM (left) and H&E-stain CFM (right). Optical signatures given by CFM, e.g. the overproliferation of cancer cells, the dissolution of normal cells by cancer, the cancer-associated adipose, the perfused and hollow vessels, and the central chaotic pathological vein with coexistence of cancer cells and erythrocytes, can also be found in the nonlinear puzzle, verifying the ability of SLAM for stain-free histopathology [5]. S: single-photon; M: multiphoton and multiharmonic. Scale bar: 500 μm.

Note S2: Two-photon FLIM measurement

One of the hallmarks of cancer is the reprogramming of energy metabolism [6], which can be analyzed by NADH and FAD lifetime [7]. However, the employment of multiphoton FLIM could result in low SNR (Figure S9) [8] and spectral crosstalk due to broadband emission. For absolute isolation of NADH and FAD in multiphoton FLIM, we will design a multispectral system to perform more dimensional analysis.

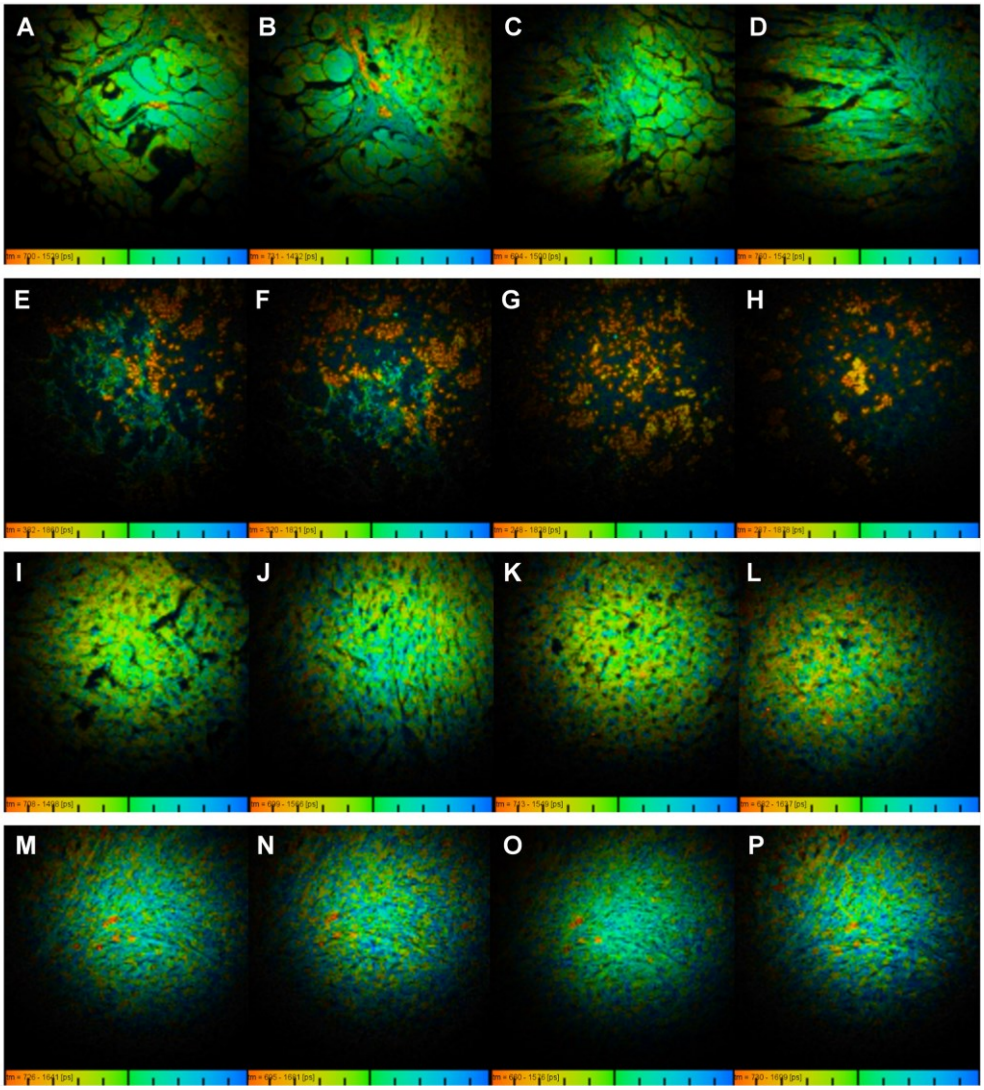

Figure S9. Two-photon FLIM images of adipose, blood, non-metastasis (hepatic) and metastatic cancer.

Note S3: Different pathological stages in livers analyzed by NADH and FAD

When using of bi-exponential fit throughout this paper, we have made sure that the thi-exponential fit did not improve χ^2 (< 50%), and the proportions of NADPH are lower than 3% of the total area of an image at any tissue constituents. We can see from Figure S10A that the cancer regions (III-C) are quite distinct from other stages (I, II and III-A) and the metastatic border is clearly observed in NADH lifetime image of III-B. The normal liver peaks the highest fluorescence lifetime, whereas the liver for *in situ* pancreatic cancer has a lower lifetime distribution (Figure S10B). The lifetime of III-B stage exhibits two peaks: the peak of higher τ_m originating from normal hepatocytes while the one of smaller τ_m originating from cancer cells. The valley between two peaks implies the transformation of two cell types. The fluorescence lifetime for the stage II are close to that of stage I, but as the tumor cells infiltrated into the hepatocytes, it decreases dramatically with an increase of acquisition time. The overall τ_m from I, II, III-A and III-C stages are 1.61 ns, 1.57 ns, 1.38 ns and 0.29 ns, respectively (Figure S10C), and are contributed by bi-component: free NADH with short lifetime and bound NADH with long lifetime [9-11]. The equilibrium between NAD+ (FAD+) and NADH (FAD) is related to the fraction of free to bound NADH (FAD), which exhibits a dramatical difference between I and III-C in Figure S10D. And the lifetime attenuation of bound NADH between cancer cells and non-metastasis hepatocytes can dramatically reach 54%. These may due to the changes in cellular metabolic pathways and in distribution of NADH enzyme binding sites caused by neoplastic hypoxia [12, 13]. Free NADH localized in the cytosol dominates the glycolysis processes ($R_{f\text{-}NADH}$ ~ 90%), rather than its bound form in mitochondria related to oxidative phosphorylation reactions.

Associated largely with oxidative phosphorylation pathway [14], FAD shifts from a longer lifetime in the free state to a shorter lifetime in a bound state [11]. The normal hepatocytes and cancer cells can be visually identified in Figure S10E. FAD shows a reverse trend of NADH in many ways, for instance, the cancer cells (instead of the control) peak the highest fluorescence lifetime (Figure S10F); with the cancerous development, the overall τ_m increases from 1.5 ns (I) to 2.0 ns (III-C) (Figure S10G) and the total intensity exhibits a substantial reduction; the lifetime of free and bound FAD at stage I are respectively 26% and 50% longer than those at stage III-C (Figure S10H). Bound FAD localized in mitochondria dominates the oxidative phosphorylation processes. It is noted that during the evolutionary process the mean contributions of bound FAD (free NADH) to the total concentration show slight fluctuation (about 73±3% for bound FAD in Figure S10H and 83±3% for free NADH in Figure S10D), this may attribute to difficulty of sole FLIM in achieving component ratios. Part of cellular respiration shifted to glycolysis, producing ATP in anaerobic conditions (free NADH) without reducing oxidative phosphorylation (bound FAD) [15].

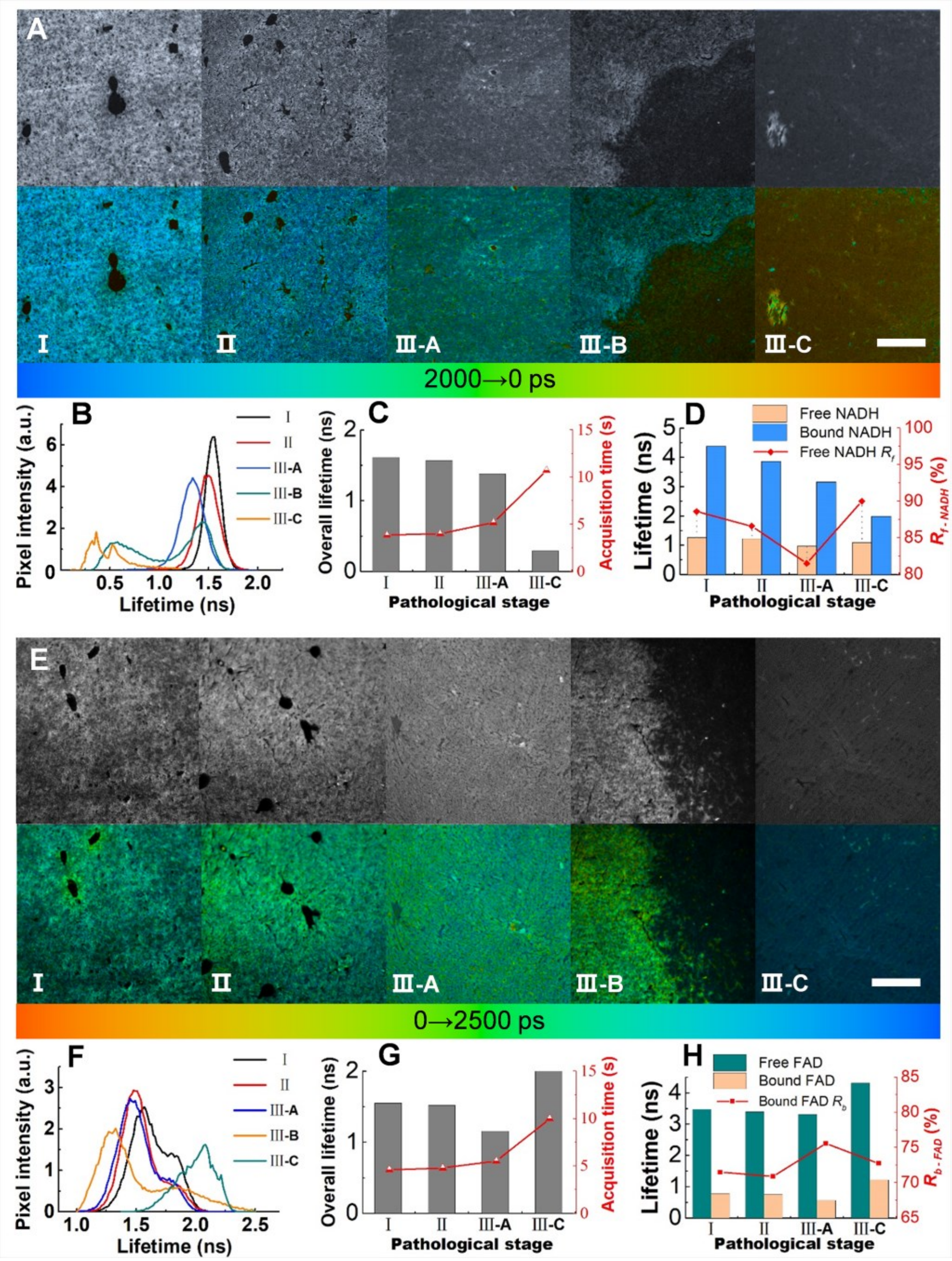

Figure S10. Different pathological stages in mouse livers analyzed by NADH (A–D) and FAD (E–H) FLIM. Pathological examination of the experimental group was performed by an experienced biomedical professor (F.Z.) during the surgery and categorized into 3 groups, one of which was further categorized into 3 subgroups as I: Control; II: Liver with *in situ* pancreatic cancer; III-A: Hepatocytes near metastasis; III-B: Boundary between non- and metastasis; III-C: metastatic cancer. **A** and **E**, Pseudo-color lifetime images. **B**, **F**, Histogram of average fluorescence life times (τ_m). **C** and **G**, Histogram of overall lifetime and the corresponding aquired time, component lifietimes and component ratios (free NADH: $R_{f\text{-}NADH}$, bound FAD: $R_{b\text{-}FAD}$) of NADH (**D**) and FAD (**H**). Scale bar: 200 μm.

Note S4: Phasor approach for component analysis.

A phasor plot representing graphically the mathematics aiming to resolve heterogeneous systems originally [16] was introduced by Jameson *et al.* [17]. For the time-correlated single-photon counting (TCSPC) technique, the decay data from each pixel of an image are transformed to the Fourier space using the following relations:

$$s_{i,j}(\omega) = \frac{\int_0^\infty I(t)\sin(n\omega t)dt}{\int_0^\infty I(t)dt} \quad (1)$$

$$g_{i,j}(\omega) = \frac{\int_0^\infty I(t)\cos(n\omega t)dt}{\int_0^\infty I(t)dt} \quad (2)$$

where i and j represent the pixel of the image, sine (s) and cosine (g) are phasor coordinates, $\omega = 2\pi f$ (f is the laser repetition rate) and n is the harmonic frequency. Independently of their location in the FLIM image, pixels with similar decay signature form clusters in the phasor plot (Figure S11).

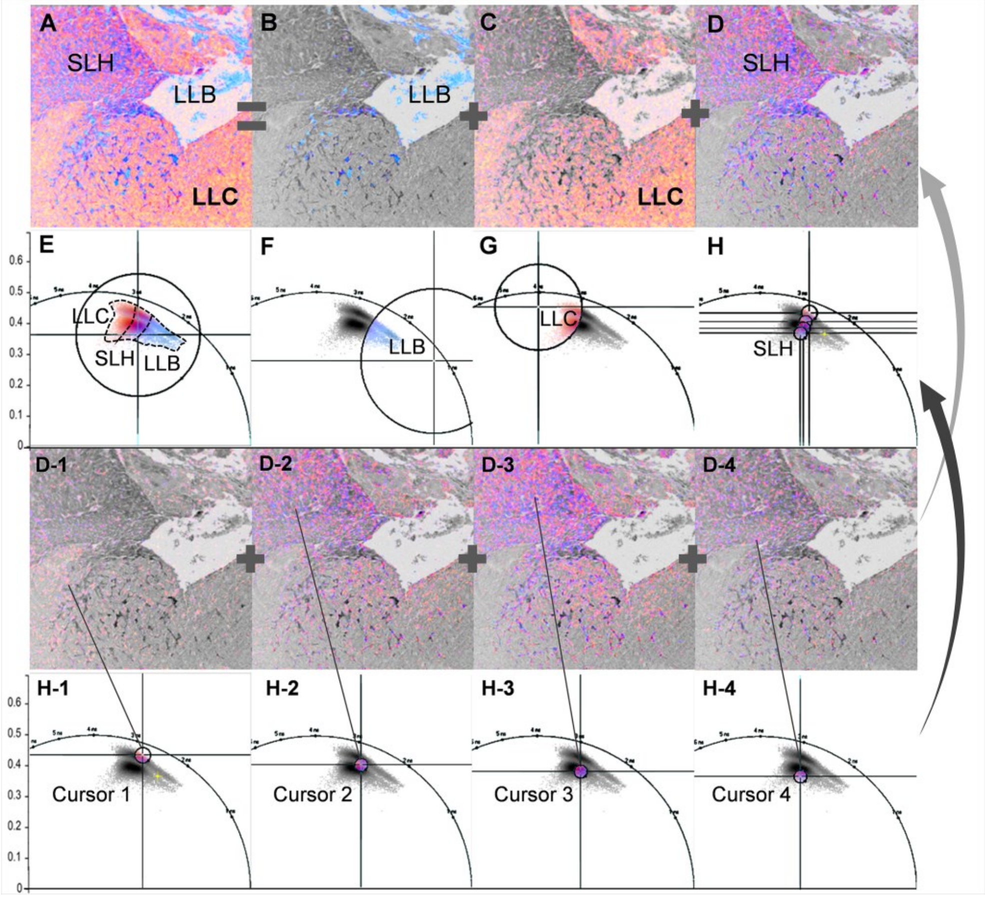

Figure S11. Pseudo-color lifetime images (A–D) and phasor plots (E–H). The group of cursors (**H**) are a combination of serial cursors (**H-1–4**), corresponding to the color-mapped lifetime images (**D-1–4**).

The ratio of free and bound FAD, and the third component (related to lipid oxidation product) are

calculated by the metabolism quantitative equations based on phasor-FLIM [11]

$$R_{f\text{-}FAD} = \frac{D_{BI}}{D_{FB}} \tag{3}$$

$$R_{b\text{-}FAD} = \frac{D_{FI}}{D_{FB}} \tag{4}$$

$$R_{third} = \frac{D_{EI}}{D_{IT}} \tag{5}$$

Where D_{ij} is distance of point i and j (i, j = F, B, T, E or I displayed in Figure S12). Note that these ratios of the free- and bound-state FAD (NADH), related to their "redox ratio", are no more than a relative measure of their concentrations.

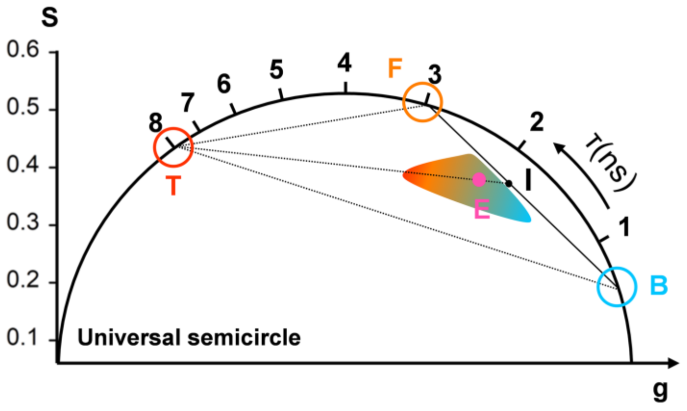

Figure S12. Expected positions and their linear combination in phasor space. F: free FAD (NADH), B: bound FAD (NADH), T: third component (if exists), E: experimental point, I: intersection point of the line of F and B and the extension line of T and E.

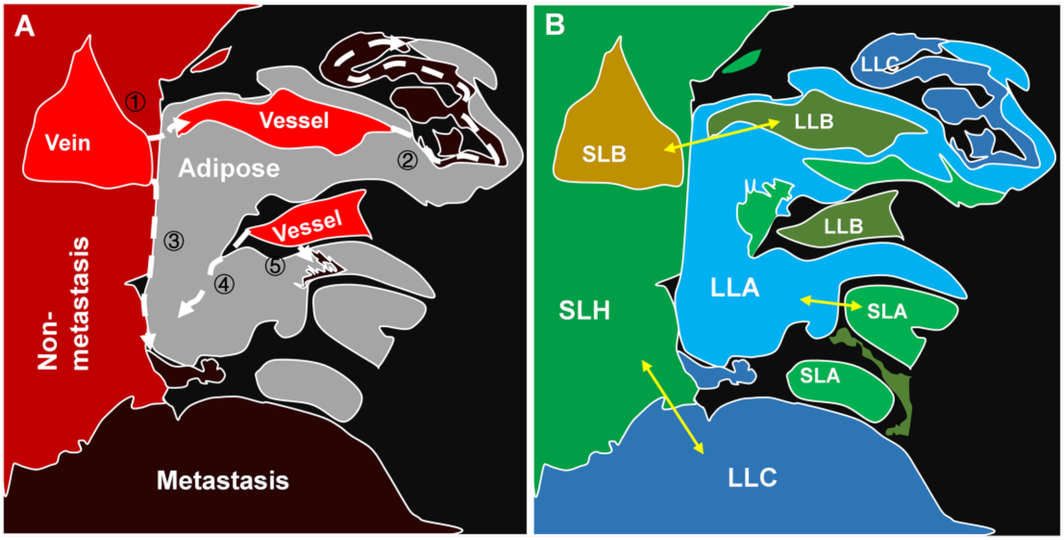

Figure S13. Thumbnails of SLAM (A) and FLIM (B) giving the major messages in a metastatic colony. White dashed arrows: invasion routes of cancer cells; yellow double-head arrows: lifetime contrast of relatively-normal and cancer-associated tissue components.

Note S5: Optical resolution

A main challenge in optical microscopy, was the limited spatial resolution set by the diffraction of light (fluorescence). Ernst Abbe first described this resolution limitation in 1873, considering objects separated by a distance smaller than approximately half of the wavelength of light, e.g. the minimum resolution for fluorescence of 400–700 nm is approximately 325–570 nm (taking into account the NA = 0.75 of the objective). Particularly, multiphoton microscopy (MPM) with longer excitation wavelength has a lower spatial resolution than that of CFM. In order to improve the resolution while maintaining the penetration depth, high NA objective, reliable PSF (point spread function) algorithm and especially, super-resolution techniques, such as saturated structured-illumination microscopy (SSIM, resolution: ~50 nm) [18], photoactivated localization microscopy (PALM, resolution: ~25 nm) [19, 20], stimulated emission depletion (STED, resolution: ~20 nm) microscopy [21, 22] and multiphoton harmonic spatial frequency modulation imaging (MP-SPIFI, resolution: ~ 362 nm) [23-25], are developed. To determine the resolved performance of our system, we estimated the Rayleigh resolution by 20-nm-diameter fluorescent beads as shown in Figure S14.

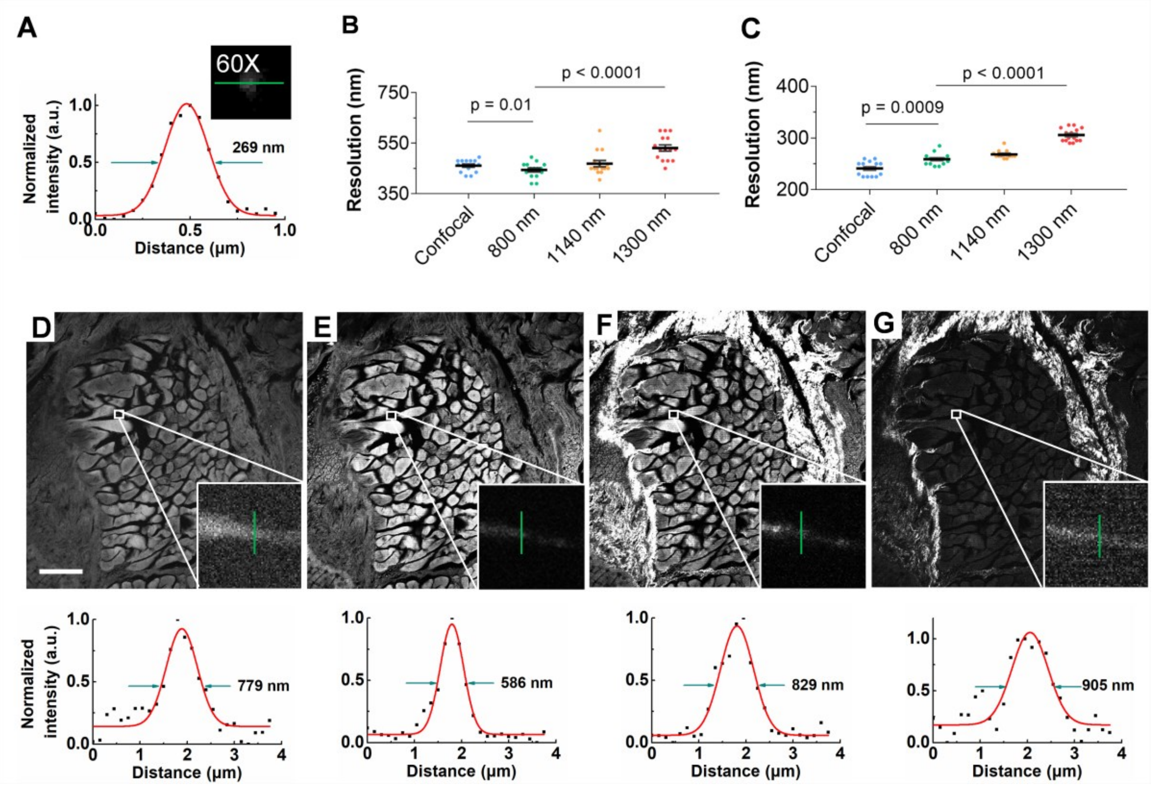

Figure S14. Evaluation of optical resolution of the system. Optical resolution measurements, using excitation wavelengths of 488 nm (CFM, FLIM), 800 nm, 1140 nm and 1300 nm (SLAM) to image 20-nm-diameter fluorescent beads (**A-C**) at a pixel resolution of 4096 × 4096, corresponding to ~150 nm per pixel, for a 0.75 NA objective; and ~50 nm per pixel, for a 1.40 NA objective. The bead's cross-sectional profiles in the lateral dimensions are shown with Gaussian fits (see Figure S14A as an example). Rayleigh resolution

values for a 0.75 NA objective (**B**) were determined as 461 ± 6 nm, n = 15 for Confocal, 444 ± 8 nm, n = 15 for 800 nm excitation, 469 ± 12 nm, n = 15 for 1140 nm excitation and 531 ± 13 nm, n = 15 for 1300 nm excitation (significant different). For a higher NA =1.40 objective the Rayleigh resolutions (**C**) were 241 ± 4 nm, n = 15 for Confocal, 259 ± 3 nm, n = 15 for 800 nm excitation, 268 ± 3 nm, n = 12 for 1140 nm excitation and 306 ± 3 nm, n = 15 for 1300 nm excitation (significant different). Mean ± s.e.m. In comparison, the FWHM of a collagen fibril in the liver tissue (**D-G**) imaged by the 20× objective was calculated as 779 nm (confocal without staining), 586 nm (800 nm excitation), 829 nm (1140 nm excitation), and 905 nm (1300 nm excitation). The resolution of the system is sufficiently high to reveal collagen fiber orientation of the cancerous zone. Scale bar: 100 μm. Unpaired two-sided Mann–Whitney test was performed between two-column data (confocal and 800 nm) and one-way ANOVA was performed between three-column data (800, 1140 and 1300 nm) in B and C.

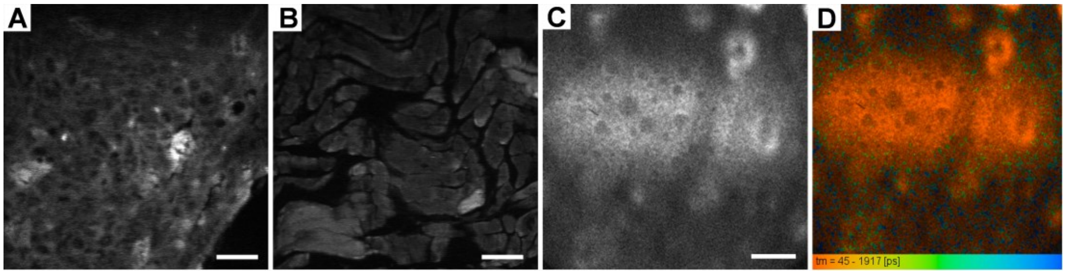

Figure S15. Investigation of sole supercontinuum picosecond laser on nonlinear and linear optical imaging. A–B, SLAM images of liver section by the supercontinuum picosecond laser at 800 nm excitation. **C–D**, Liver tissue by single-photon FLIM. Scale bar: 100 μm.

Table S1 Specified dichroic mirrors and bandpass filters for excitation wavelength of 1080 nm.

Laser (nm)	Group	LP (nm)	BP (nm)	Modality	Object
1080	A	488	450/50	3PA	NADH
				3PA	**FAD
		593	540/10	SHG	Collagen
				2PA	*FAD
				3PA	**FAD
		685	641/75	2PA	FAD
				2PA	**NADH
	B	495	360/10	THG	Heterogeneity

* marks concomitant signal which can be filtered out by the mathematical function (subtract) in ImageJ

** marks the negligible signal as compared to the main signal in the same channel.

Table S2 Specified dichroic mirrors and bandpass filters for excitation wavelength of 1300 nm.

Laser (nm)	Group	LP (nm)	BP (nm)	Modality	Object
1300	A	488	420/60	THG	Heterogeneity
		593	550/88	3PA	FAD
		685	650/10	SHG	Collagen
				3PA	*FAD
			700/75	2PA	FAD

Table S3 Specified dichroic mirrors and bandpass filters for excitation wavelength of 1140 nm.

Laser (nm)	Group	LP (nm)	BP (nm)	Modality	Object
1140	A	488	450/50	3PA	NADH
				3PA	**FAD
		593	570/10	SHG	Collagen
				2PA	*FAD
				3PA	**FAD
		685	641/75	2PA	FAD
				2PA	**NADH
	B	495	380/10	THG	Heterogeneity
		560	525/50	3PA	FAD
				3PA	**NADH

Supplementary References

1. Paduch R. The role of lymphangiogenesis and angiogenesis in tumor metastasis. Cell Oncol. 2016; 39: 397–410.
2. J. KS, H. BC, Y. K. The pathogenesis of cancer metastasis: relevance to therapy. Dordrecht: Springer; 2009.
3. Valastyan S, Weinberg RA. Tumor Metastasis: Molecular Insights and Evolving Paradigms. Cell. 2011; 147: 275–92.
4. Deng T, Lyon CJ, Bergin S, Caligiuri MA, Hsueh WA. Obesity, Inflammation, and Cancer. Annu Rev Pathol-Mech. 2016; 11: 421–49.
5. Tu HH, Liu Y, Turchinovich D, Marjanovic M, Lyngso JK, Laegsgaard J, et al. Stain-free histopathology by programmable supercontinuum pulses. Nat Photonics. 2016; 10: 534.
6. Hanahan D, Weinberg RA. Hallmarks of Cancer: The Next Generation. Cell. 2011; 144: 646–74.
7. Meleshina AV, Dudenkova VV, Bystrova AS, Kuznetsova DS, Shirmanova MV, Zagaynova EVJSCR, et al. Two-photon FLIM of NAD(P)H and FAD in mesenchymal stem cells undergoing either osteogenic or chondrogenic differentiation. Stem Cell Res Ther. 2017; 8: 15.
8. Wang HL, Liang XW, Mohammed YH, Thomas JA, Bridle KR, Thorling CA, et al. Real-time histology in liver disease using multiphoton microscopy with fluorescence lifetime imaging. Biomed Opt Express. 2015; 6: 780–92.
9. Zhang ZH, Li H, Liu Q, Zhou LL, Zhang M, Luo QM, et al. Metabolic imaging of tumors using intrinsic and extrinsic fluorescent markers. Biosens Bioelectron. 2004; 20: 643–50.
10. Skala MC, Riching KM, Gendron-Fitzpatrick A, Eickhoff J, Eliceiri KW, White JG, et al. In vivo multiphoton microscopy of NADH and FAD redox states, fluorescence lifetimes, and cellular morphology in precancerous epithelia. P Natl Acad Sci USA. 2007; 104: 19494–9.
11. Ranjit S, Malacrida L, Jameson DM, Grafton E. Fit-free analysis of fluorescence lifetime imaging data using the phasor approach. Nat Protoc. 2018; 13: 1979.
12. Fenderson, Bruce. Molecular Biology of the Cell,5th Edition. Shock. 2008; 30: 100.
13. Banerjee S, Bhatt DK. Histochemical studies on the distribution of certain dehydrogenases in squamous cell carcinoma of cheek. Indian Journal of Cancer. 1989; 26: 21.
14. Heikal AA. Intracellular coenzymes as natural biomarkers for metabolic activities and mitochondrial anomalies. Biomark Med. 2010; 4: 241–63.
15. Thies S, Hong Y, Viglianti BL, Cathryn P, Shubha A, Zeljko V, et al. Spatial heterogeneity and oxygen dependence of glucose consumption in R3230Ac and fibrosarcomas of the Fischer 344 rat. Cancer Res. 2005; 65: 5163–71.
16. Weber G. Resolution of the fluorescence lifetimes in a heterogeneous system by phase and modulation measurements. Journal of Physical Chemistry. 1980; 85: 949–53.
17. Jameson D, EnricoGratton, Hall RJASR. The Measurement and Analysis of Heterogeneous Emissions by Multifrequency Phase and Modulation Fluorometry. 1983; 20: 55–106.
18. Gustafsson MGL. Nonlinear structured-illumination microscopy: Wide-field fluorescence imaging with theoretically unlimited resolution. P Natl Acad Sci USA. 2005; 102: 13081-6.
19. Betzig E, Patterson GH, Sougrat R, Lindwasser OW, Olenych S, Bonifacino JS, et al. Imaging intracellular fluorescent proteins at nanometer resolution. Science. 2006; 313: 1642-5.

20. Betzig E. Proposed Method for Molecular Optical Imaging. Optics Letters. 1995; 20: 237-9.
21. Hell SW. Far-field optical nanoscopy. Science. 2007; 316: 1153-8.
22. Hell SW. Toward fluorescence nanoscopy. Nat Biotechnol. 2003; 21: 1347-55.
23. Field JJ, Wernsing KA, Domingue SR, Motz AMA, DeLuca KF, Levi DH, et al. Superresolved multiphoton microscopy with spatial frequency-modulated imaging. P Natl Acad Sci USA. 2016; 113: 6605-10.
24. Schlup P, Futia G, Bartels RA. Lateral tomographic spatial frequency modulated imaging. Appl Phys Lett. 2011; 98.
25. Hoover EE, Field JJ, Winters DG, Young MD, Chandler EV, Speirs JC, et al. Eliminating the scattering ambiguity in multifocal, multimodal, multiphoton imaging systems. J Biophotonics. 2012; 5: 425-36.
